# Anti-TNF Treatment Response in Rheumatoid Arthritis Patients Is Associated with Genetic Variation in the NLRP3-Inflammasome

**DOI:** 10.1371/journal.pone.0100361

**Published:** 2014-06-26

**Authors:** Jacob Sode, Ulla Vogel, Steffen Bank, Paal Skytt Andersen, Marianne Kragh Thomsen, Merete Lund Hetland, Henning Locht, Niels H. H. Heegaard, Vibeke Andersen

**Affiliations:** 1 Clinical Biochemistry, Immunology & Genetics, Statens Serum Institut, Copenhagen, Denmark; 2 Department of Rheumatology, Frederiksberg Hospital, Frederiksberg, Denmark; 3 Institute of Regional Health Research, University of Southern Denmark, Odense, Denmark; 4 National Research Centre for the Working Environment, Copenhagen, Denmark; 5 Department of Medicine, Viborg Regional Hospital, Viborg, Denmark; 6 Biomedicine, University of Aarhus, Aarhus, Denmark; 7 Department of Microbiology and Infection Control, Statens Serum Institut, Copenhagen, Denmark; 8 Department of Clinical Microbiology, Aarhus University Hospital, Aarhus, Denmark; 9 The DANBIO Registry, Copenhagen Center for Arthritis Research, Center for Rheumatology and Spine Diseases, Glostrup Hospital, Glostrup, Denmark; 10 Department of Clinical Medicine, Faculty of Health and Medical Sciences, University of Copenhagen, Copenhagen, Denmark; 11 Organ Center, Hospital of Southern Jutland, Aabenraa, Denmark; 12 OPEN (Odense Patient data Explorative Network), Odense University Hospital, Odense, Denmark; University of Michigan, United States of America

## Abstract

**Objective:**

Many patients with rheumatoid arthritis (RA) benefit from tumor necrosis factor-α blocking treatment (anti-TNF), but about one third do not respond. The objective of this study was to replicate and extend previously found associations between anti-TNF treatment response and genetic variation in the TNF-, NF-κB- and pattern recognition receptor signalling pathways.

**Methods:**

Forty-one single nucleotide polymorphisms (SNPs), including 34 functional, in 28 genes involved in inflammatory pathways were assessed in 538 anti-TNF naive Danish RA patients with clinical data. Multivariable logistic regression analyses were performed to test associations between genotypes and treatment response at 3–6 months using the *European League Against Rheumatism* (EULAR) response criterion. American College of Rheumatology treatment response (ACR50) and relative change in 28-joint disease activity score (relDAS28) were used as secondary outcomes. Subgroup analyses were stratified according to smoking status, type of anti-TNF drug and IgM-Rheumatoid Factor (IgM-RF) status. False discovery rate (FDR) controlling was used to adjust for multiple testing.

**Results:**

Statistically significant associations with EULAR response were found for two SNPs in *NLRP3*(rs4612666) (OR (odds ratio) for good/moderate response = 0.64 (95% confidence interval: 0.44–0.95), p = 0.025, q = 0.95) and *INFG*(rs2430561) (OR = 0.40 (0.21–0.76), p = 0.005, q = 0.18) and among IgM-RF positive patients for *TNFRS1A*(rs4149570) (0.59 (0.36–0.98), p = 0.040, q = 0.76). Current smokers who carried the *NLRP3*(rs4612666) variant allele were less likely to benefit from anti-TNF treatment (OR = 0.24 (0.10–0.56), p = 0.001, q = 0.04).

**Conclusions:**

In a population of Danish RA patients, we confirm the *NLRP3* gene as associated with EULAR anti-TNF response as previously reported. The *NLRP3* variant (T) allele is associated with lower treatment response, in particular among current smokers. Furthermore, we find that a functional polymorphism in the interferon-γ gene is associated with anti-TNF response. All findings should be tested by replication in independent validation cohorts and augmented by assessing cytokine levels and activities of the relevant gene products.

## Introduction

Tumour necrosis factor α inhibitors (anti-TNF) have improved the treatment of rheumatoid arthritis (RA); however, the effect varies and approximately one third of patients do not respond [1]. Furthermore, the anti-TNF drugs are expensive and have potentially severe side effects including adverse immunological reactions and increased risk of serious infections [2]. Thus, biomarkers predictive of anti-TNF treatment outcome are likely to improve treatment of patients with RA.

Accordingly, several studies have evaluated biochemical markers, which alone or in combination with clinical parameters are associated with treatment response [3], [4]. Until now, a locus in the *PTPRC* gene that was first identified in a genome-wide association study (GWAS) [5], [6] is the only confirmed genetic marker that can predict treatment response [7], [8]. Recently, polymorphisms in genes encoding the NLRP3-inflammasome (*NLRP3, CARD8*) [9], nuclear factor κ B (NF-κB) and Toll-like receptor (TLR) signalling pathways (*TLR2, TLR4, MyD88, CHUK*) [10] have been associated with *European League Against Rheumatism* (EULAR) response to anti-TNF treatment.

Anti-TNF treatment blocks TNF from binding to TNF receptor 1 (TNFR1), thereby preventing NF-κB signaling. It seems likely that genetic variants in genes encoding proteins in the inflammatory pathways involved in NF-κB activation are involved in the differential response to anti-TNF drugs.

NF-κB is a central regulator of inflammation and regulates the expression of more than 150 genes (http://www.bu.edu/nf-kb/gene-resources/target-genes/) including *TNFA* (TNF-α), *TNFAIP3* (A20), *TLR2*, *TLR9*, *CD14*, *NFKBIA* (IκBα), *NFKB1* (NF-κB1/p50-RelA), *IL1B*, *IL1RN*, *IL6*, *IL10*, *IL17A* and *IFNG*.

The *NLRP3* gene (cryopyrin/NALP3) encodes one of several proteins forming the NLRP3-inflammasome, an intracellular innate immune sensor that also includes the cysteine protease, caspase-1. Upon inflammasome activation by cellular stress and damage [11], caspase-1 controls activation and cellular release of IL-1β and IL-18, both strong pro-inflammatory cytokines.

NF-κB activation [12] as well as increased TLRs [13] and NLRP3 [14] expression can be detected in synovial tissue from RA patients, but several other molecules activating the pathway (TNF-α, IL-1β) [15] or regulated by the pathway, e.g. IL-17 [15], are central in the pathogenesis of RA.

The objective of this study was to replicate and extend the reported associations between anti-TNF treatment response and genes in the TNF-, NF-κB- and pattern recognition receptor signalling pathways (TLRs and NLRP3). We assessed 41 mainly functional polymorphisms based on available knowledge to allow a biological interpretation of associations with treatment response to anti-TNF, thus potentially adding new knowledge on the underlying causes of treatment success and failure. We used the nationwide Danish database with prospectively collected clinical data of patients with RA (the DANBIO registry).

## Methods

### Ethics Statement

The study was conducted in accordance with the Declaration of Helsinki and was approved by the Regional Ethics Committee of Central Denmark Region (M-20100153 and S-20120113) and the Danish Data Protection Agency (J. 2010-41-4719). The Regional Ethics Committee of Central Denmark Region gave exemption from informed consent requirements because samples were taken for other reasons and data were not identifiable.

### Patients and samples

The DANBIO registry includes data on patients with inflammatory joint diseases, monitored prospectively as part of routine care during treatment with synthetic and biologic disease-modifying anti-rheumatic drugs (DMARDs) [16]. We linked the clinical data from DANBIO with blood samples available from the routine screening for tuberculosis performed prior to treatment with anti-TNF agents.

We included 538 anti-TNF naïve patients with RA in the study. All these patients initiated their first anti-TNF treatment, had clinical variables registered at baseline and follow-up in DANBIO, and had blood samples available.

Biological material (after whole blood analysis for *Mycobacterium tuberculosis* infection) was collected at Statens Serum Institut from September 2009 and at Aarhus University Hospital from January 2011 and until 1^st^ of July 2012.

### Candidate gene analyses

We chose to focus on genes involved in the NF-κB, TNF-α and pattern recognition receptor signalling pathways (see full list of polymorphisms in Table S1). Candidate polymorphisms in genes in these pathways were identified by searching for “polymorphism AND Gene name AND (reporter gene OR luciferase OR ELISA OR RT-PCR OR flow cytometry OR EMSA)” in PubMed in August 2011 [17], [18]. After an extensive literature search approximately 100 polymorphisms were found and 41 were subsequently chosen primarily based on evidence of biological effect and secondly based on association with autoimmune disease. We screened for linkage disequilibrium (LD) in SNAP, a web-based database based on the International HapMap Project [19]. The selected polymorphisms had expected minor allele frequencies (MAF) ranging from 5% to 48%.

The polymorphisms were genotyped by PCR-based KASP genotyping assay by KBioscience (KBioscience, Hoddesdon, United Kingdom - www.lgcgenomics.com) on extracted DNA (Maxwell 16 LEV Blood DNA Kit, Promega, Madison, Wisconsin, USA) as described by Bank et al. [20]. Genotyping of *TNF* (TNF-α) rs1799724 and rs1800630 failed due to their close proximity to each other, causing bias in genotype failure. All other chosen assays had a call rate exceeding 97%.

### Outcome measures, data handling, and statistical methods

Patient data from the DANBIO database included pre-treatment and follow-up data on tender and swollen joint counts (28 joints), C-reactive protein (CRP, mg/l) and patient global score on a 100 mm VAS-scale, which were combined to calculate the disease activity score (DAS28) [21]. Furthermore, data on baseline DMARD treatment (yes/no), type of anti-TNF drug, IgM rheumatoid factor (IgM-RF) status (ICD-10: seropositive RA DM5.9/seronegative RA DM6.0+DM6.9), sex, age, smoking status (never/previous/current) and functional status assessed by the health assessment questionnaire (HAQ, range: 0–3, with 3 being completely disabled), were also drawn from DANBIO. Titers on IgM-RF and anti-citrullinated protein antibodies (ACPA) were not available.

The baseline (pre-treatment) visit was defined as a visit 0–30 days before start of anti-TNF treatment and follow-up as a visit within 60–180 days after treatment onset (at the contact closest to 120 days if more than one visit was registered).

Primary outcome was EULAR response criteria (good, moderate or none) at follow-up [21].

Univariate statistical analysis was used to assess the association between EULAR good/moderate response and genotypes under a dominant model (chi-square tests). Multivariable logistic regression analyses were then performed to investigate association between EULAR good or moderate response and genotype with the variant alleles grouped together (dominant model) and, furthermore, the individual genotypes with the homozygote wildtype as the reference genotype.

The final model included the following baseline covariates: sex, age, HAQ, CRP, baseline DMARD and IgM-RF status. Smoking was not included in the final analyses as data were missing for 15% of patients. However, in patients with available data, we assessed gene-smoking interaction for all polymorphisms by stratifying patients into current-, previous- and never smokers. We also stratified patients into anti-TNF subgroups to check drug specific associations. The remaining variables were also checked for interaction with the polymorphisms using Wald’s test and likelihood-ratio test.

Secondary outcomes were the relative change in DAS28 (*relDAS28 = (baseline DAS28-follow-upDAS28)/baseline DAS28*) and the American College of Rheumatology outcome measure, ACR50 response. Furthermore, we used ACR70 response in case of statistically significant association for ACR50 response and gene variants. Multivariate linear regression analysis was performed for relDAS28.

All statistical analyses were performed in Stata version 12 (StataCorp, Texas, USA). The Genetic Power Calculator was used for power analysis of discrete traits [22].

At the 5% significance level and MAF of 0.1, 0.25 and 0.4 we had >80% power to detect effect sizes of 1.5, 1.5 and 1.5, respectively, assuming a dominant genetic model. For a recessive model the corresponding effect sizes were 2.9, 1.8 and 1.6, respectively.

We performed correction for multiple testing using False Discovery Rate (FDR) classical one-stage method set at 0.05 (q-value) [23].

## Results

### Study population

Clinical and demographic baseline characteristics of the 538 patients are presented in Table 1. The patients were treated with the following anti-TNF drugs: infliximab (31.2%), etanercept (30.9%), adalimumab (24.9%), golimumab (9.1%) or certolizumab (3.9%). Overall, EULAR responses (good, moderate, and none) were achieved for 42.9%, 27.5% and 29.6%, respectively.

**Table 1:** Baseline clinical and demographic characteristics.

	All RA patients	Seropositive RA patients	Seronegative RA patients
No.	538 (−)	407 (75.7%)	131 (24.3%)
Female	407 (75.7%)	303 (74.4%)	105 (80.2%)
Age/years (SD)			
at treatment start	55.0 (13.0)	55.7 (12.8)	53.0 (13.6)
Smoking status			
Current	142 (31.8%)	110 (32.7%)	32 (29.1%)
Previous	151 (33.9%)	120 (35.7%)	31 (28.2%)
Never	153 (34.3%)	106 (31.6%)	47 (42.7%)
Missing data	92 (−)	71 (−)	21 (−)
DMARD	453 (84.2%)	343 (84.3%)	110 (84.0%)
VAS Patient global score (0–100)/Mean (SD)	62.6 (22.6)	60.8 (22.9)	68.5 (20.7)
VAS Physician global score (0–100)/Mean (SD)	38.4 (20.7)	38.0 (20.2)	39.8 (22.3)
VAS pain score (0–100)/Mean (SD)	58.0 (22.8)	55.9 (23.2)	64.4 (20.2)
TJC 0-28/Mean (SD)	9.5 (7.3)	9.0 (7.0)	11.1 (8.1)
SJC 0-28/Mean (SD)	5.4 (4.6)	5.6 (4.5)	4.8 (4.8)
HAQ score/Mean (SD)	1.2 (0.7)	1.2 (0.7)	1.3 (0.7)
CRP/mg/mL (SD)	19.7 (25.5)	20.5 (27.0)	17.2 (20.3)
DAS28/mean (SD)	4.9 (1.2)	4.8 (1.2)	5.0 (1.1)
Anti-TNF drug			
Infliximab (%)	168 (31.2%)	122 (30.0%)	46 (35.1%)
Etanercept (%)	166 (30.8%)	124 (30.5%)	42 (32.1%)
Adalimumab (%)	134 (24.9%)	105 (25.8%)	29 (22.1%)
Golimumab (%)	49 (9.1%)	38 (9.3%)	11 (8.4%)
Certolizumab (%)	21 (3.9%)	18 (4.4%)	3 (2.3%)
EULAR response			
Good (%)	231 (42.9%)	178 (43.7%)	53 (40.5%)
Moderate (%)	148 (27.5%)	108 (26.5%)	40 (30.5%)
None (%)	159 (29.6%)	121 (29.7%)	38 (29.0%)
ACR50 response (%)	170 (31.6%)	131 (32.2%)	39 (29.8%)
RelDAS28 response (SD)	0.28 (0.32)	0.28 (0.34)	0.285 (0.27)

SD: standard deviation, DMARD: disease modifying anti-rheumatic drugs, VAS: visual analogue scale, TJC: tender joint count, SJC: swollen joint count, HAQ: health assessment score, CRP: C-reactive protein, DAS28: disease activity score (28-joints), EULAR: European League Against Rheumatism, ACR50: American College of Rheumatology, 50% improvement, RelDAS28: relative change in DAS28.

### Primary outcome EULAR response

Statistically significant associations with EULAR response were found for polymorphisms in *NLRP3* (rs4612666), *IFNG* (rs2430561) and *TNFRSF1A* (rs4149570) (Table 2, Table S2).

**Table 2:** Genotypes of associated polymorphisms and adjusted odds ratios for associations between gene variants and EULAR anti-TNF treatment response.

								EULAR GOOD/MODERATE			EULAR GOOD					
*GENE*(SNP)	GENOTYPE		FREQ.	NONE	MODERATE	GOOD		ADJ.OR	95% CI	P-VALUE			ADJ. OR		95% CI	P-VALUE
All RA																
*IFNG*	TT	137		34	37		66	Ref.				Ref.				
rs2430561	TA	263		74	71		118	0.81	(0.50–1.31)	0.395		0.75		(0.45–1.27)		0.285
	AA	114		40	38		36	0.59	(0.34–1.02)	0.059		0.40		(0.21–0.76)		0.005**
	TA or AA	377		114	109		154	0.73	(0.47–1.15)	0.177		0.63		(0.38–1.03)		0.067
*NLRP3*	CC	275		69	84		122	Ref.				Ref.				
rs4612666	CT	210		73	54		83	0.62	(0.42–0.92)	0.018*		0.62		(0.40–0.97)		0.037*
	TT	31		9	8		14	0.85	(0.37–1.96)	0.707		0.89		(0.36–2.24)		0.808
	CT or TT	241		82	62		97	0.64	0.44–0.95)	0.025*		0.65		(0.43–1.00)		0.050*
**Seropositive RA**																
*IFNG*	TT	106		26	30		50	Ref.				Ref.				
rs2430561	TA	205		56	55		94	0.85	(0.49–1.46)	0.547		0.83		(0.43–1.51)		0.544
	AA	78		30	21		27	0.51	(0.26–0.96)	0.038*		0.42		(0.20–0.87)		0.020*
	TA or AA	283		86	76		121	0.73	(0.43–1.22)	0.229		0.69		(0.39–1.21)		0.196
*NLRP3*	CC	212		51	62		99	Ref.				Ref.				
rs4612666	CT	156		55	39		62	0.58	(0.37–0.92)	0.020*		0.58		(0.35–0.96)		0.035*
	TT	25		9	5		11	0.59	(0.24–1.43)	0.241		0.64		(0.24–1.71)		0.375
	CT or TT	181		64	44		73	0.58	(0.37–0.90)	0.016*		0.59		(0.36–0.96)		0.032*
*TNFRSF1A*	GG	137		33	40		64	Ref.				Ref.				
rs4149570	GT	196		68	47		81	0.59	(0.36–0.98)	0.040*		0.63		(0.37–1.09)		0.102
	TT	56		15	18		23	0.89	(0.43–1.85)	0.760		0.82		(0.37–1.82)		0.619
	GT or TT	252		83	65		104	0.65	(0.40–1.04)	0.074		0.67		(0.39–1.13)		0.130

Logistic regression, adjusted (Adj.) for sex, age, HAQ, CRP, DMARD at baseline, IgM RF status (seropositive/seronegative). CI: confidence interval, Freq.: Frequency, OR: odds ratio, EULAR: European League Against Rheumatism, P-value: *<0.05, **<0.01.

**Figure 1:**
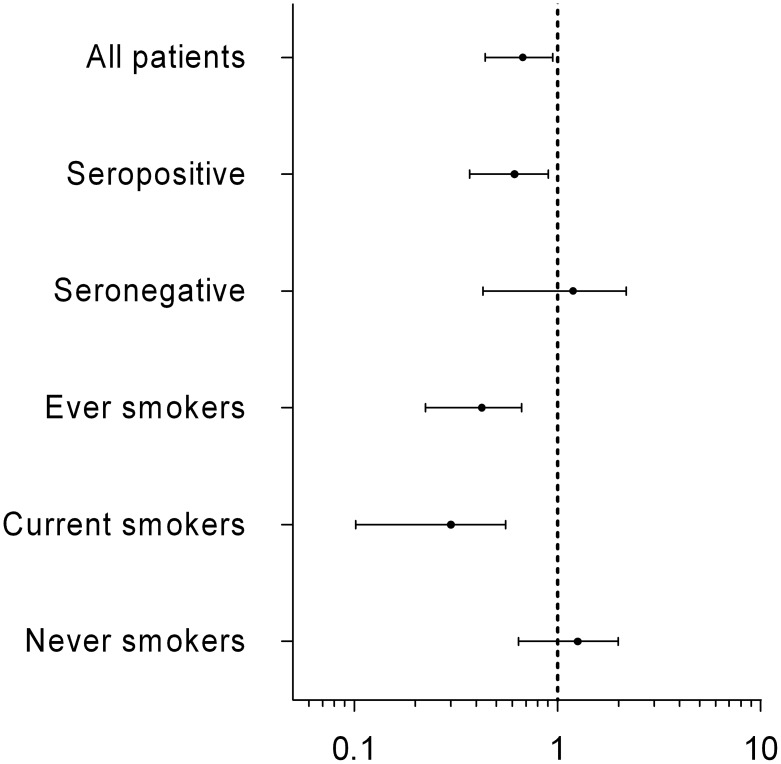
Odds ratio (OR) for association between *NLRP3* (rs4612666) variant allele and EULAR good/moderate response (log scale, 95% confidence interval). Patients stratified on diagnose (seropositive-/seronegative RA) or smoking status. Smoking as independent predictor of EULAR good/moderate response: OR = 1.018, p = 0.941.

*NLRP3* rs4612666 variant allele carriers had a significantly lower chance of achieving EULAR good/moderate response (OR = 0.64, p = 0.025, q = 0.95) and EULAR good response (OR = 0.65, p = 0.050, q = 0.97). Stratification according to both smoking status and to IgM RF status revealed even lower OR among seropositive RA patients and current smokers (Table 2, Figure 1).

For *IFNG* rs2430561, the homozygous variant genotype was significantly associated with a lower chance of EULAR good response (OR = 0.40, p = 0.005, q = 0.18), and–among seropositive RA patients–with a lower chance of both EULAR good/moderate response (OR = 0.51, p = 0.038, q = 0.83) and EULAR good response (OR = 0.42, p = 0.020, q = 0.70).

*TNFRSF1A* rs4149570 was associated with EULAR good/moderate response (OR = 0.59, p = 0.040, q = 0.760) in the seropositive subgroup analysis, exclusively.

### Secondary outcomes relDAS28 and ACR50

Of the three polymorphisms associated with EULAR response, *NLRP3* rs4612666 (seropositive RA: p = 0.024, q = 0.65) and *TNFRSF1A* rs4149570 (seropositive RA: p = 0.034, q = 0.65) were also significantly associated with the relDAS28 (Table 3, Table S3a–b).

**Table 3:** Adjusted odds ratio (OR) for associations between gene variants and ACR50 and relDAS28 response to anti-TNF treatment.

			ACR50 response						relDAS28		
*GENE* (SNP)	GENOTYPE	Freq.	No	Yes	Adj. OR	95% CI	P-VALUE		Adj. Coeff.	95% CI	P-VALUE
ALL RA											
*IL1B*	GG	255	181	74							
rs1143623	GC	223	149	74	1.22	(0.82–1.80)	0.330		0.02	(−0.04–0.08)	0.489
	CC	37	20	17	2.14	(1.05–4.35)	0.037*		0.02	(−0.09–0.13)	0.693
	GC/CC	260	169	91	1.32	(0.91–1.93)	0.145		0.02	(−0.04–0.08)	0.466
*TLR4*	GG	253	183	70							
rs5030728	GA	215	134	81	1.58	(1.06–2.35)	0.023*		0.01	(−0.05–0.07)	0.786
	AA	46	32	14	1.21	(0.61–2.42)	0.584		0.01	(−0.09–0.11)	0.794
	GA/AA	261	166	95	1.51	(1.03–2.21)	0.033*		0.01	(−0.05–0.06)	0.748
*NLRP3*	CC	275	183	92							
rs4612666	CT	210	147	63	0.86	(0.58–1.27)	0.434		−0.06	(−0.12–0.00)	0.049*
	TT	31	21	10	0.97	(0.44–2.17)	0.947		−0.04	(−0.16–0.08)	0.524
	CT/TT	241	168	73	0.87	(0.60–1.27)	0.467		−0.06	(−0.11–0.00)	0.050*
**SEROPOSITIVE RA**											
*IL17A*	GG	176	127	49							
rs2275913	GA	172	106	66	1.73	(1.09–2.75)	0.021*		0.01	(−0.06–0.08)	0.752
	AA	44	30	14	1.30	(0.63–2.69)	0.471		0.05	(−0.06–0.16)	0.363
	GA/AA	216	136	80	1.63	(1.05–2.54)	0.030*		0.02	(−0.05–0.09)	0.562
*NLRP3*	CC	212	137	75							
rs4612666	CT	156	109	47	0.80	(0.51–1.25)	0.330		−0.08	(−0.15–0.01)	0.024*
	TT	25	17	8	0.90	(0.37–2.19)	0.813		−0.08	(−0.22–0.06)	0.256
	CT/TT	181	126	55	0.81	(0.53–1.25)	0.345		−0.08	(−0.15–0.01)	0.018*
*TLR4*	TT	147	90	57							
rs12377632	TC	189	133	56	0.64	(0.40–1.02)	0.061		−0.03	(−0.10–0.04)	0.400
	CC	50	36	14	0.59	(0.29–1.21)	0.152		−0.02	(−0.13–0.09)	0.708
	TC/CC	239	169	70	0.63	(0.41–0.98)	0.042*		−0.03	(−0.10–0.04)	0.412
*TNFRSF1A*	GG	137	87	50							
rs4149570	GT	196	137	59	0.77	(0.48–1.23)	0.268		−0.08	(−0.15–0.01)	0.034*
	TT	56	38	18	0.81	(0.41–1.60)	0.544		−0.04	(−0.14–0.07)	0.506
	GT/TT	252	175	77	0.78	(0.50–1.21)	0.267		−0.07	(−0.14–0.00)	0.051

Adj. OR: adjusted odds ratio for ACR50 and coefficient (Coeff.) for relative change in DAS28 (relDAS28). Adjusted for gender, age, HAQ-, DMARD at baseline, CRP, IgM RF status (seropositive/seronegative). Freq.: frequency.

Additionally, significant associations with ACR50 outcome (Table 3, Table S3a-c) were found for variant allele carriers of *TLR4* rs5030728 (OR = 1.51, p = 0.033, q = 0.82) and for carriers of the homozygote variant genotype of *IL1B* rs1143623 (OR = 2.14, p = 0.037, q = 0.90) among all RA patients.

Among seropositive RA patients, the variant alleles of *TLR4* rs12377632 (OR = 0.63, p = 0.042, q = 0.61) and *IL17A* rs2275913 (OR = 1.63, p = 0.030, q = 0.61) were significantly associated with ACR*5*0 response.

Statistically significant associations were found between ACR70 response (13.9% of patients) and *IL1B* (rs1143623, rs1143627), and *IL17A* (rs2275913) but not *IL1B* (rs4848306) and *TLR4* (rs12377632, rs1554973, rs5030728) (data not shown).

### Smoking and anti-TNF drug stratified analyses

Subgroup analyses stratifying for smoking status and type of anti-TNF drug was performed (Table S4+S5).

Among current smokers, the variant alleles of *NLRP3* rs4612666 (OR = 0.24, p = 0.001, q = 0.04) and *IL4R* rs1805010 (OR = 2.69, p = 0.028, q = 0.56) were significantly associated with EULAR good/moderate response.

In patients treated with infliximab three polymorphisms (*IL1B* (rs4848306) (OR = 2.84, p = 0.004, q = 0.16), *LY96* (rs11465996) (OR = 2.21, p = 0.023, q = 0.45), *NLRP3* (rs4612666) (OR = 0.50, p = 0.041, 0.53)) were associated with EULAR good/moderate response. For etanercept-treated patients, the variant allele of *TNFRSF1A* rs4149570 (OR = 0.40, p = 0.025, q = 0.87) was associated with EULAR good/moderate response. No drug-specific association with any polymorphism was found for adalimumab-treated patients. However, in the seropositive subgroup significant associations with polymorphisms were found for infliximab (*TNF*), etanercept (*LY96, NFKB1, NLRP3, TNFRSF1A*) and adalimumab (*IL23R, CD14, TLR5*) treated patients.

No associations were found for the combined group of anti-TNF drugs based on monoclonal antibodies (all except etanercept).

### Summarized effect of polymorphisms

**Figure 2:**
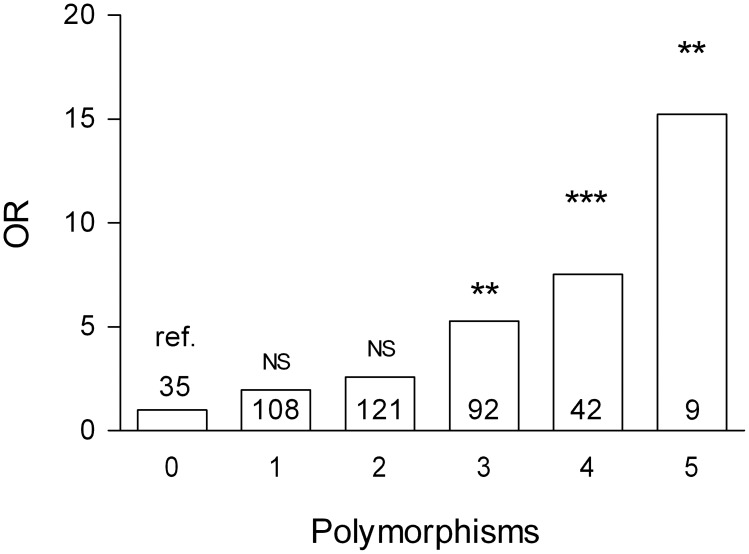
Aggregate genetic risk score. Weighted odds ratio (OR) for seropositive RA patients’ risk of EULAR non-response according to the number of associated polymorphisms, relative to patients with zero associated polymorphisms. Number of patients in bars. NS: not significant. P-value: **<0.01, ***<0.001.

For each seropositive RA patient we counted the alleles (homozygote wildtype or carrier of variant) that were significantly associated with EULAR non-response and calculated a weighted aggregated genetic risk score. The risk of EULAR non response increased *per* associated polymorphism, and patients with five polymorphisms associated had about 15 times higher risk of EULAR non response than did patients with no associated alleles (Figure 2).

## Discussion

We assessed the associations between 41 inflammatory pathway-related polymorphisms and the response to treatment with anti-TNF in 538 anti-TNF naive RA patients in a genetically homogeneous and clinically well-characterised and closely monitored cohort of RA patients [7]. The polymorphisms were chosen in genes encoding proteins in the TNF-, NFκB- and pattern recognition receptor signalling pathways (TLRs and NLRP3). Most of the chosen polymorphisms (34/41) were functional, *i.e.,* affecting gene expression or protein function and, thus, allowing biological interpretation of the results.

We analysed treatment response using both EULAR response criteria, relative change in DAS28, and ACR50/ACR70. This enables comparison with other studies.

We were able to confirm the association between the *NLRP3* gene and EULAR anti-TNF response recently reported in a well-powered study on UK RA patients [9]. However, in the UK study the specific *NLRP3* polymorphism (rs4612666) was not reported as associated with EULAR response but three other polymorphisms in *NLRP3* were. Although these three polymorphisms are poor markers for rs4612666 (r^2^ for linkage with rs4612666: rs4925659, r^2^ = 0.005; rs10925026, r^2^ = 0.09; rs4925648, r^2^ = 0.22), there is relatively strong linkage disequilibrium between rs4612666 and rs10925026 (D’ = 0.81) (Figure S1). Thus, two different polymorphisms in an area of the *NLRP3* gene with low recombination have now been associated EULAR good and moderate response vs no response in two different cohorts treated with anti-TNF. This is a strong indicator of a true association of the gene with treatment response. As indicated, there are contradicting results for rs4612666 between our and the UK study [9]. This may be due to a type 1 error in our study. On the other hand it can also reflect differences in the population of the two cohorts, e.g. the proportion of the specific anti-TNF drugs used for treatment is different in the two studies. Also, geographical/ethnic variation may play a role. Further validation is needed to clear this question.

The data also showed that a polymorphism in *IFNG*–and in subgroups, polymorphisms *IL1B, LY96, TLR2* and *TNFRSF1A*–were significantly associated with EULAR response to anti-TNF treatment. However, due to the low number of observations in subgroups and the fact that they, to our knowledge, have not earlier been linked to a differentiated anti-TNF response, these results should be considered preliminary.

When analysing the secondary outcome (ACR50), polymorphisms in *IL1B, IL17* and *TLR4* were associated with response to anti-TNF treatment. Another study [10] also found a polymorphism in *TLR4* associated with anti-TNF response, though this study used the EULAR response criterion.

The importance of the NLRP3-inflammasome in RA pathogenesis is illustrated by the findings of increased *NLRP3* mRNA in synovial tissue from RA patients compared to subjects with osteoarthritis [14]. Moreover, another polymorphism in *NLRP3* has been found to be associated with delayed apoptosis of neutrophils suggesting that NLRP3 could influence the resolution of inflammation through a dysregulated innate immune response [24].

Hitomi *et al*. found the rs4612666 variant allele to cause lower mRNA expression by decreasing the transcriptional enhancer activity (in intron 7) of the *NLRP3* gene in a cell expression study [25].

It is premature to give a solid biological interpretation on the association found for *NLRP3* (rs4612666) as the association of this specific polymorphism has not been replicated. However, since the association found in this polymorphism remained statistically significant after correction for multiple testing (among smoking variant allele carriers) it is tempting to hypothesize that this SNP is functionally relevant for the outcome of anti-TNF treatment in RA patients.

Smoking produce exogenous reactive oxygen species (ROS) [26] and ROS has been found to increase expression and activation of the NLRP3-inflammasome [27]. This may explain why the largest ORs were found in the subgroup of current smokers in whom increased activation of a differentially expressed NLRP3-inflammasome should have the strongest biological effects (carriers of the *NLRP3* variant (T) allele were less likely to benefit from anti-TNF treatment). Taken together, our results indicate a potential impact of smoking on anti-TNF response and suggest that the non-responders may have lower expression of *NLRP3* (Table 4).

**Table 4:** Polymorphisms’ effect on anti−/pro-inflammatory signal and chance of good anti-TNF response (EULAR good/moderate or ACR50).

*GENE/*PROTEIN (SNP)	ProteinInflammatoryeffect	Variant allele Expectedeffect on geneexpression	Variant alleleAssociation withAnti-TNF response	Subgroup
*NLRP3*/NLRP3	Pro-	↓[25]	↓*	All
rs4612666 C>T				Smokers
				Seropositive
				Infliximab
*TNFRS1A*/TNFR1	Pro-	↑[32]	↓*	Seropositive
rs4149570 G>T				Etanercept
*IL17A*/IL-17	Pro-	↑[33]	↑#	Seropositive
rs2275913 G>A				
*TLR4/*TLR4	Pro-	Unknown	↑#	All
*rs5030728* G>A				
*IL1B/*IL-1β	Pro-	↑[34]	↑#	All
rs1143623 G>C				Seropositive
*IL1B/*IL-1β	Pro-	↓[34]	↑*	Infliximab
rs4848306				
*LY96*/MD-2	Pro-	↑[35]	↑*	Infliximab
rs11465996 C>G				
*INFG*/Interferon-γ	Pro−/Anti-	↓[36]	↓*	All
rs2430561 T>A				Seropositive

EULAR (*): European League Against Rheumatism ACR50 (#): American College of Rheumatology response criterion (50% improvement).

This study was not designed to detect effect sizes below 1.5 and had low power to perform subgroup analyses. In the seropositive subgroup there was >80% power to detect effect sizes above 1.7 for polymorphisms with MAFs above 0.25, whereas power in the seronegative subgroup was too low for relevant analyses. Though also limited by low power we found it relevant to analyse associations for individual drugs and monoclonal-based anti-TNFs because of the small differences and similarities which exist between them.

We have chosen to present significant result at the 5% significance level. Though most of these results become insignificant after correction for multiple testing, we also present FDR corrected q-value. We cannot exclude that choosing this level of statistical significance may result in type 1 errors but obviously may too conservative testing result in type 2 errors [28]. Due to the exploratory nature of the study we found it relevant to present even weak genetic associations, which may subsequently be replicated in other studies. Other factors than significance level should be considered when interpreting these results [29]. The prior probability of finding associations in functional polymorphisms is higher since earlier studies have found these to result in an altered expression of their corresponding genes. These genes were chosen based on their expected biological effects in the mechanism of action of the anti-TNF treatment.

Also the presence or absence of systematic bias is important for the risk of type 1 error. In this study, the selection of patients was based on the availability of sufficient clinical data to calculate EULAR responses and this could theoretically create a selection bias. Overall, however, the response rates found in this study are comparable to on the response rates in the complete DANBIO cohort [30] and other comparable studies [10], [31]. Genotyping artefacts are theoretically possible and were identified for two polymorphisms but re-genotyping of 94 randomly selected samples in another cohort yielded >99% identical genotypes.

A strength of the study is that the clinical data were collected independently and prospectively as part of routine care.

In conclusion, we confirm the *NLRP3* gene as associated with anti-TNF treatment response based on EULAR criteria in a Danish cohort of RA patients [9]. The *NLRP3* variant (T) allele is associated with a poorer treatment response, in particular among current smokers. Furthermore, we find the homozygous variant genotype of an *IFNG* polymorphism associated with a poorer anti-TNF response.

Results indicating association with other polymorphisms among subgroups in this cohort should be interpreted with care and all findings tested by replication in independent validation cohorts.

## Supporting Information

Figure S1 **Linkage Disequilibrium Plot for polymorphisms spanning the** ***NLRP3*** **-gene. D’ values are shown.** Darker grey indicates higher r^2^.
(PNG)

Table S1 **Chosen polymorphisms and corresponding gene.** Associated effect of polymorphism.
(DOCX)

Table S2 **Adjusted odds ratios for associations between gene variants and EULAR anti-TNF treatment response.** (a. All RA patients, b. Seropositive RA patients).
(DOCX)

Table S3 **Adjusted odds ratio (OR)/coefficient for associations between gene variants and ACR50 and relDAS28 response to anti-TNF treatment.** (a. All RA patients, b. Seropositive RA patients).
(DOCX)

Table S4 **Smoking strata: Odds ratio for minor allele carriers achieving EULAR good/moderate response**.
(DOCX)

Table S5 **Anti-tumour necrosis factor (TNF) subgroup odds ratio for EULAR good/moderate response.** (a. All RA patients, b. Seropositive RA patients).
(DOCX)
