# Supplementary figures and images for: Anti-TNF Treatment Response in Rheumatoid Arthritis Patients Is Associated with Genetic Variation in the NLRP3-Inflammasome
Source: PLoS One. 2014 Jun 26;9(6):e100361. doi: 10.1371/journal.pone.0100361 (PMC4072633; doi:10.1371/journal.pone.0100361)

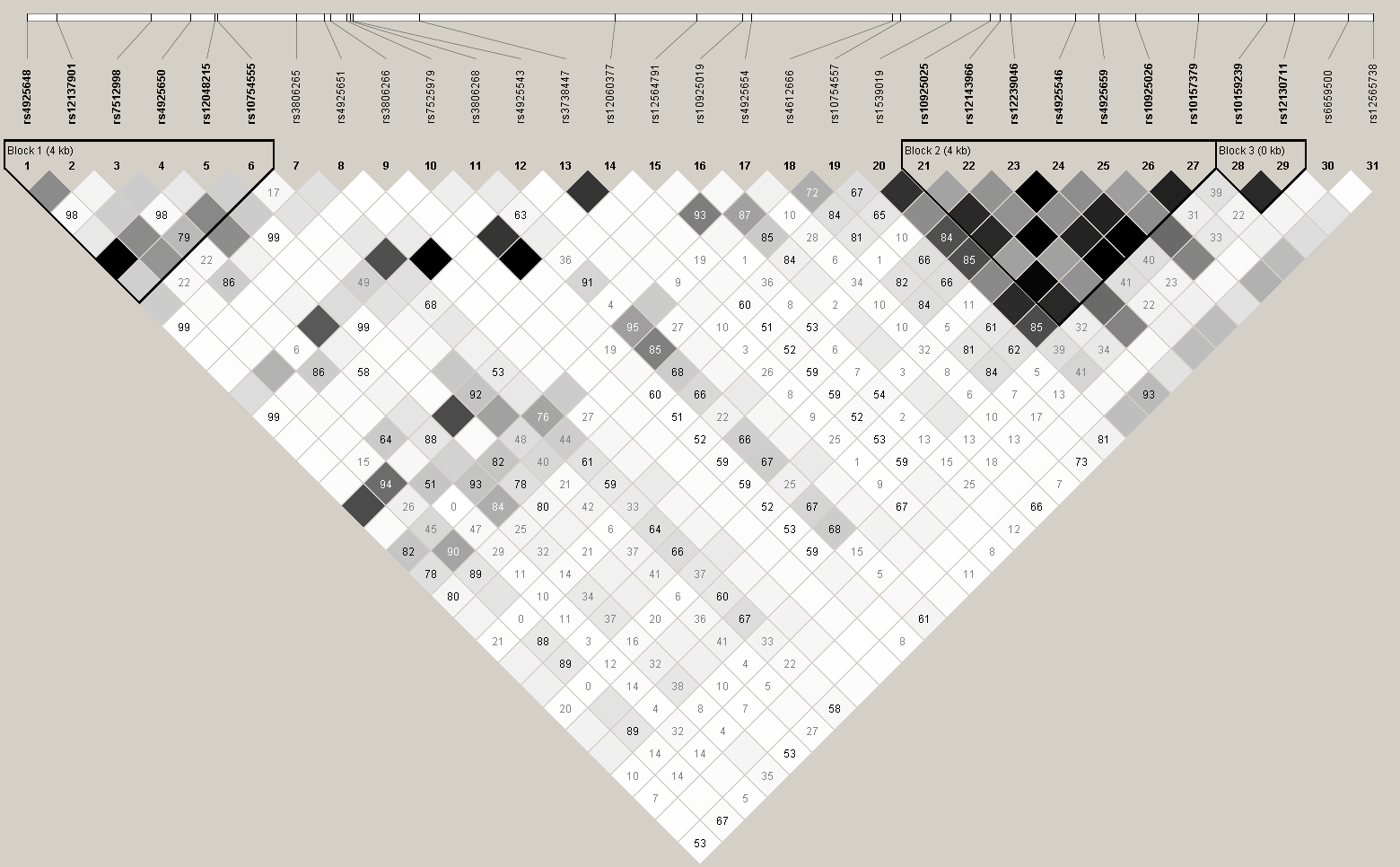

Supplement: Figure S1 — Linkage Disequilibrium Plot for polymorphisms spanning the NLRP3 -gene. D’ values are shown. Darker grey indicates higher r2. (PNG) [file pone.0100361.s001.png]
