# Supplementary material for: Anti-TNF Treatment Response in Rheumatoid Arthritis Patients Is Associated with Genetic Variation in the NLRP3-Inflammasome
Source: PLoS One. 2014 Jun 26;9(6):e100361. doi: 10.1371/journal.pone.0100361 (PMC4072633; doi:10.1371/journal.pone.0100361)
Supplement: Table S1 — Chosen polymorphisms and corresponding gene. Associated effect of polymorphism. (DOCX) [file pone.0100361.s002.docx]

| **Supplementary Table 1.** Chosen polymorphisms and corresponding gene. Associated effect of polymorphism. | | | | | |  |
| --- | --- | --- | --- | --- | --- | --- |
| GENE | | GENETIC VARIANT | WILDTYPE/ VARIANT | NCBI GENE ID | MAF* | VARIANT ASSOCIATED EFFECTS |
| *CD14* | | rs2569190 | G/A | 929 | 47.4 | increased CD14 level (AA) [1] |
| *IFNG* | | rs2430561 | T/A | 3458 | 43.3 | Decreased IFN- γ level (A) [2] |
| *IL1B* | | rs1143623 | G/C | 3553 | 32.8 | Increased expression (C)† [3,4] |
| *IL1B* | | rs1143627 | T/C | 3553 | 36.3 | Increased expression (C)† [3–5] |
| *IL1B* | | rs4848306 | G/A | 3553 | 46.4 | Decreased transcription (A) [4,6] |
| *IL1RN* | | rs4251961 | T/C | 3557 | 38.5 | Decreased IL-1RA level (C) [7] |
| *IL4R* | | rs1805010 | A/G | 3566 | 49.1 | Decreased IL-17 level (A) [8] |
| *IL6* | | rs10499563 | T/C | 3569 | 19.5 | Decreased expression (C) [9] |
| *IL6R* | | rs4537545 | C/T | 3570 | 36.8 | Increased IL-6r and IL-6 level (TT) [10] |
| *IL10* | | rs1800872 | C/A | 3586 | 20.8 | Increased serum IL-10 levels (T) [11] |
| *IL10* | | rs3024505 | C/T | 3586 | 18.1 | Unknown |
| *IL17A* | | rs2275913 | G/A | 3605 | 35.4 | Increased IL-17production (A) [12] |
| *IL23R* | | rs11209026 | G/A | 149233 | 4.1 | Decreased IL-17 production (A) [13] |
| *LY96* | | rs11465996 | C/G | 23643 | 35.8 | Increased LY96 (MD-2) & TNF-α level (G) [14] |
| *MAP3K14* | | rs7222094 | T/C | 9020 | 40.8 | Decreased CXCL10 protein levels (CC) [15] |
| *NFKB1* | | rs28362491 | ATTG/del | 4790 | 34.0  ** | Increased expression (ATTG ins.) [16] |
| *NFKBIA* | | rs17103265 | T/del | 4792 | 5.6  *** | Decreased expression (del) [17] |
| *NFKBIA* | | rs696 | G/A | 4792 | 38.9 | Increased expression (A) [18] |
| *NLRP3* | | rs4612666 | C/T | 114548 | 23.0 | Decreased expression (T) [19] |
| *PPARG* | | rs1801282 | C/G | 5468 | 9.7 | Decreased expression (G) [20] |
| *PTPN22* | | rs2476601 | G/A | 26191 | 11.7 | Decreased TNF-α in serum (A) [21] |
| *SUMO4* | | rs237025 | T/C | 387082 | 50.0 | Increased NFκB1 expression(C) [22] |
| *TGFB1* | | rs1800469 | C/T | 7040 | 28.8 | Increased TGF-beta serum levels (TT) [23] |
| *TLR2* | | rs11938228 | C/A | 7097 | 29.6 | Unknown [24] |
| *TLR2* | | rs1816702 | C/T | 7097 | 14.2 | Increased receptor level (T) [25] |
| *TLR2* | | rs3804099 | T/C | 7097 | 45.1 | Decreased TNF-α, IL-1β & IL-6 levels (CC). Increased MyD88 expression (CC) [26] |
| *TLR2* | | rs4696480 | T/A | 7097 | 46.0  *** | Unknown [24] |
| *TLR4* | | rs12377632 | T/C | 7099 | 36.7 | Unknown [24] |
| *TLR4* | | rs1554973 | T/C | 7099 | 21.7 | Unknown [24] |
| *TLR4* | | rs5030728 | G/A | 7099 | 30.5 | Unknown [24] |
| *TLR5* | | rs5744168 | C/T | 7100 | 5.1  **** | Reduced TNF-α, IL-1β & IL-6 level [26] and inhibited TLR5 function (T) [27] |
| *TLR9* | | rs187084 | T/C | 54106 | 34.1 | Decreased expression (C) [28] |
| *TLR9* | | rs352139 | G/A | 54106 | 48.2 | Increased expression (A) [28] |
| *TNF* | | rs1799724 | C/T | 7124 | 6.7 | Increased TNF-α level(T) [29] |
| *TNF* | | rs1800629 | G/A | 7124 | 17.3 | Increased expression(A) [30] |
| *TNF* | | rs1800630 | C/A | 7124 | 15.0 | Increased expression(A) [31] |
| *TNF* | | rs361525 | G/A | 7124 | 5.1  *** | Decreased expression (A) [32] |
| *TNFAIP3* | | rs6927172 | C/G | 7128 | 17.5 | Increased expression(G) [33] |
| *TNFRSF1A* | | rs1800693 | A/G | 7132 | 48.2 | Soluble ∆6-TNFR1 [34] |
| *TNFRSF1A* | | rs4149570 | G/T | 7132 | 31.4 | Increased expression (T) [35] |
| *TNFRSF10A* | | rs20575 | G/C | 8797 | 40.8 | Unknown biological effect. Associated with anti-TNF response.[36] |
| *MAF: HapMap-CEU if not stated other. **CAU200: Panel of 200 Caucasian, *** NCBI global population, ****CSAgilent: Panel of 662 of European descent in ClinSeq project. †In haplotype context with the dominating haplotype among Caucasian. | | | | | | |

Reference List

1. Mertens J, Bregadze R, Mansur A, Askar E, Bickeboller H, Ramadori G, Mihm S (2009) Functional impact of endotoxin receptor CD14 polymorphisms on transcriptional activity. J Mol Med (Berl) 87: 815-824. 10.1007/s00109-009-0479-7 [doi].

2. Kim K, Park SY, Kim T, Kang YM, Shim SC, Suh CH, Park YB, Kim CS, Kang C, Bae SC (2011) Replicated association of a regulatory polymorphism in the interferon gamma gene with lupus susceptibility. Ann Rheum Dis 70: 1878-1879. ard.2010.147249 [pii];10.1136/ard.2010.147249 [doi].

3. Wen AQ, Gu W, Wang J, Feng K, Qin L, Ying C, Zhu PF, Wang ZG, Jiang JX (2010) Clinical relevance of IL-1beta promoter polymorphisms (-1470, -511, and -31) in patients with major trauma. Shock 33: 576-582. 10.1097/SHK.0b013e3181cc0a8e [doi].

4. Chen H, Wilkins LM, Aziz N, Cannings C, Wyllie DH, Bingle C, Rogus J, Beck JD, Offenbacher S, Cork MJ, Rafie-Kolpin M, Hsieh CM, Kornman KS, Duff GW (2006) Single nucleotide polymorphisms in the human interleukin-1B gene affect transcription according to haplotype context. Hum Mol Genet 15: 519-529. ddi469 [pii];10.1093/hmg/ddi469 [doi].

5. Lind H, Haugen A, Zienolddiny S (2007) Differential binding of proteins to the IL1B -31 T/C polymorphism in lung epithelial cells. Cytokine 38: 43-48. S1043-4666(07)00102-0 [pii];10.1016/j.cyto.2007.05.001 [doi].

6. Yoshida M, Shiroiwa K, Mouri K, Ishiguro H, Supriyanto I, Ratta-Apha W, Eguchi N, Okazaki S, Sasada T, Fukutake M, Hashimoto T, Inada T, Arinami T, Shirakawa O, Hishimoto A (2012) Haplotypes in the expression quantitative trait locus of interleukin-1beta gene are associated with schizophrenia. Schizophr Res 140: 185-191. S0920-9964(12)00348-9 [pii];10.1016/j.schres.2012.06.031 [doi].

7. Rafiq S, Stevens K, Hurst AJ, Murray A, Henley W, Weedon MN, Bandinelli S, Corsi AM, Guralnik JM, Ferruci L, Melzer D, Frayling TM (2007) Common genetic variation in the gene encoding interleukin-1-receptor antagonist (IL-1RA) is associated with altered circulating IL-1RA levels. Genes Immun 8: 344-351. 6364393 [pii];10.1038/sj.gene.6364393 [doi].

8. Wallis SK, Cooney LA, Endres JL, Lee MJ, Ryu J, Somers EC, Fox DA (2011) A polymorphism in the interleukin-4 receptor affects the ability of interleukin-4 to regulate Th17 cells: a possible immunoregulatory mechanism for genetic control of the severity of rheumatoid arthritis. Arthritis Res Ther 13: R15. ar3239 [pii];10.1186/ar3239 [doi].

9. Smith AJ, D'Aiuto F, Palmen J, Cooper JA, Samuel J, Thompson S, Sanders J, Donos N, Nibali L, Brull D, Woo P, Humphries SE (2008) Association of serum interleukin-6 concentration with a functional IL6 -6331T>C polymorphism. Clin Chem 54: 841-850. clinchem.2007.098608 [pii];10.1373/clinchem.2007.098608 [doi].

10. Rafiq S, Frayling TM, Murray A, Hurst A, Stevens K, Weedon MN, Henley W, Ferrucci L, Bandinelli S, Corsi AM, Guralnik JM, Melzer D (2007) A common variant of the interleukin 6 receptor (IL-6r) gene increases IL-6r and IL-6 levels, without other inflammatory effects. Genes Immun 8: 552-559. 6364414 [pii];10.1038/sj.gene.6364414 [doi].

11. Wang AH, Lam WJ, Han DY, Ding Y, Hu R, Fraser AG, Ferguson LR, Morgan AR (2011) The effect of IL-10 genetic variation and interleukin 10 serum levels on Crohn's disease susceptibility in a New Zealand population. Hum Immunol 72: 431-435.

12. Espinoza JL, Takami A, Nakata K, Onizuka M, Kawase T, Akiyama H, Miyamura K, Morishima Y, Fukuda T, Kodera Y, Nakao S (2011) A genetic variant in the IL-17 promoter is functionally associated with acute graft-versus-host disease after unrelated bone marrow transplantation. PLoS One 6: e26229. 10.1371/journal.pone.0026229 [doi];PONE-D-11-15752 [pii].

13. Oosting M, ter HH, van de Veerdonk FL, Sturm P, Kullberg BJ, van der Meer JW, Netea MG, Joosten LA (2011) Role of interleukin-23 (IL-23) receptor signaling for IL-17 responses in human Lyme disease. Infect Immun 79: 4681-4687. IAI.05242-11 [pii];10.1128/IAI.05242-11 [doi].

14. Gu W, Shan YA, Zhou J, Jiang DP, Zhang L, Du DY, Wang ZG, Jiang JX (2007) Functional significance of gene polymorphisms in the promoter of myeloid differentiation-2. Ann Surg 246: 151-158. 10.1097/01.sla.0000262788.67171.3f [doi];00000658-200707000-00023 [pii].

15. Thair SA, Walley KR, Nakada TA, McConechy MK, Boyd JH, Wellman H, Russell JA (2011) A single nucleotide polymorphism in NF-kappaB inducing kinase is associated with mortality in septic shock. J Immunol 186: 2321-2328. jimmunol.1002864 [pii];10.4049/jimmunol.1002864 [doi].

16. Park JY, Farrance IK, Fenty NM, Hagberg JM, Roth SM, Mosser DM, Wang MQ, Jo H, Okazaki T, Brant SR, Brown MD (2007) NFKB1 promoter variation implicates shear-induced NOS3 gene expression and endothelial function in prehypertensives and stage I hypertensives. Am J Physiol Heart Circ Physiol 293: H2320-H2327. 00186.2007 [pii];10.1152/ajpheart.00186.2007 [doi].

17. Wang S, Zhang M, Zeng Z, Tian L, Wu K, Chu J, Fan D, Hu P, Sung JJ, Yu J (2011) IkappaBalpha polymorphisms were associated with increased risk of gastric cancer in a southern Chinese population: a case-control study. Life Sci 88: 792-797. S0024-3205(11)00091-9 [pii];10.1016/j.lfs.2011.02.016 [doi].

18. Song S, Chen D, Lu J, Liao J, Luo Y, Yang Z, Fu X, Fan X, Wei Y, Yang L, Wang L, Wang J (2011) NFkappaB1 and NFkappaBIA polymorphisms are associated with increased risk for sporadic colorectal cancer in a southern Chinese population. PLoS One 6: e21726. 10.1371/journal.pone.0021726 [doi];PONE-D-11-05326 [pii].

19. Hitomi Y, Ebisawa M, Tomikawa M, Imai T, Komata T, Hirota T, Harada M, Sakashita M, Suzuki Y, Shimojo N, Kohno Y, Fujita K, Miyatake A, Doi S, Enomoto T, Taniguchi M, Higashi N, Nakamura Y, Tamari M (2009) Associations of functional NLRP3 polymorphisms with susceptibility to food-induced anaphylaxis and aspirin-induced asthma. J Allergy Clin Immunol 124: 779-785.

20. Aoyagi Y, Nagata S, Kudo T, Fujii T, Wada M, Chiba Y, Ohtsuka Y, Yamashiro Y, Shimizu T, Ohkusa T (2010) Peroxisome proliferator-activated receptor gamma 2 mutation may cause a subset of ulcerative colitis. Pediatr Int 52: 729-734. PED3195 [pii];10.1111/j.1442-200X.2010.03195.x [doi].

21. Kariuki SN, Crow MK, Niewold TB (2008) The PTPN22 C1858T polymorphism is associated with skewing of cytokine profiles toward high interferon-alpha activity and low tumor necrosis factor alpha levels in patients with lupus. Arthritis Rheum 58: 2818-2823. 10.1002/art.23728 [doi].

22. Guo D, Li M, Zhang Y, Yang P, Eckenrode S, Hopkins D, Zheng W, Purohit S, Podolsky RH, Muir A, Wang J, Dong Z, Brusko T, Atkinson M, Pozzilli P, Zeidler A, Raffel LJ, Jacob CO, Park Y, Serrano-Rios M, Larrad MT, Zhang Z, Garchon HJ, Bach JF, Rotter JI, She JX, Wang CY (2004) A functional variant of SUMO4, a new I kappa B alpha modifier, is associated with type 1 diabetes. Nat Genet 36: 837-841. 10.1038/ng1391 [doi];ng1391 [pii].

23. Grainger DJ, Heathcote K, Chiano M, Snieder H, Kemp PR, Metcalfe JC, Carter ND, Spector TD (1999) Genetic control of the circulating concentration of transforming growth factor type beta1. Hum Mol Genet 8: 93-97. ddc008 [pii].

24. Gast A, Bermejo JL, Claus R, Brandt A, Weires M, Weber A, Plass C, Sucker A, Hemminki K, Schadendorf D, Kumar R (2011) Association of Inherited Variation in Toll-Like Receptor Genes with Malignant Melanoma Susceptibility and Survival. PLoS ONE 6: e24370. doi:10.1371/journal.pone.0024370.

25. Bielinski SJ, Hall JL, Pankow JS, Boerwinkle E, Matijevic-Aleksic N, He M, Chambless L, Folsom AR (2011) Genetic variants in TLR2 and TLR4 are associated with markers of monocyte activation: the Atherosclerosis Risk in Communities MRI Study. Hum Genet 129: 655-662. 10.1007/s00439-011-0962-4 [doi].

26. Zhang F, Gao XD, Wu WW, Gao Y, Zhang YW, Wang SP (2013) Polymorphisms in toll-like receptors 2, 4 and 5 are associated with Legionella pneumophila infection. Infection . 10.1007/s15010-013-0444-9 [doi].

27. Hawn TR, Verbon A, Lettinga KD, Zhao LP, Li SS, Laws RJ, Skerrett SJ, Beutler B, Schroeder L, Nachman A, Ozinsky A, Smith KD, Aderem A (2003) A common dominant TLR5 stop codon polymorphism abolishes flagellin signaling and is associated with susceptibility to legionnaires' disease. J Exp Med 198: 1563-1572. 10.1084/jem.20031220 [doi];jem.20031220 [pii].

28. Tao K, Fujii M, Tsukumo S, Maekawa Y, Kishihara K, Kimoto Y, Horiuchi T, Hisaeda H, Akira S, Kagami S, Yasutomo K (2007) Genetic variations of Toll-like receptor 9 predispose to systemic lupus erythematosus in Japanese population. Ann Rheum Dis 66: 905-909. ard.2006.065961 [pii];10.1136/ard.2006.065961 [doi].

29. Lv K, Chen R, Cai Q, Fang M, Sun S (2006) Effects of a single nucleotide polymorphism on the expression of human tumor necrosis factor-alpha. Scand J Immunol 64: 164-169. SJI1786 [pii];10.1111/j.1365-3083.2006.01786.x [doi].

30. Karimi M, Goldie LC, Cruickshank MN, Moses EK, Abraham LJ (2009) A critical assessment of the factors affecting reporter gene assays for promoter SNP function: a reassessment of -308 TNF polymorphism function using a novel integrated reporter system. Eur J Hum Genet 17: 1454-1462.

31. Udalova IA, Richardson A, Denys A, Smith C, Ackerman H, Foxwell B, Kwiatkowski D (2000) Functional consequences of a polymorphism affecting NF-kappaB p50-p50 binding to the TNF promoter region. Mol Cell Biol 20: 9113-9119.

32. Kaluza W, Reuss E, Grossmann S, Hug R, Schopf RE, Galle PR, Maerker-Hermann E, Hoehler T (2000) Different transcriptional activity and in vitro TNF-alpha production in psoriasis patients carrying the TNF-alpha 238A promoter polymorphism. J Invest Dermatol 114: 1180-1183. jid001 [pii];10.1046/j.1523-1747.2000.00001.x [doi].

33. Marotte H, Miossec P (2010) Biomarkers for prediction of TNFalpha blockers response in rheumatoid arthritis. Joint Bone Spine 77: 297-305. S1297-319X(10)00090-4 [pii];10.1016/j.jbspin.2010.02.026 [doi].

34. Gregory AP, Dendrou CA, Attfield KE, Haghikia A, Xifara DK, Butter F, Poschmann G, Kaur G, Lambert L, Leach OA, Promel S, Punwani D, Felce JH, Davis SJ, Gold R, Nielsen FC, Siegel RM, Mann M, Bell JI, McVean G, Fugger L (2012) TNF receptor 1 genetic risk mirrors outcome of anti-TNF therapy in multiple sclerosis. Nature 488: 508-511. 10.1038/nature11307.

35. Wang GB, Li CR, Yang J, Wen PQ, Jia SL (2011) A regulatory polymorphism in promoter region of TNFR1 gene is associated with Kawasaki disease in Chinese individuals. Hum Immunol 72: 451-457. S0198-8859(11)00032-2 [pii];10.1016/j.humimm.2011.02.004 [doi].

36. Morales-Lara MJ, Canete JD, Torres-Moreno D, Hernandez MV, Pedrero F, Celis R, Garcia-Simon MS, Conesa-Zamora P (2012) Effects of polymorphisms in TRAILR1 and TNFR1A on the response to anti-TNF therapies in patients with rheumatoid and psoriatic arthritis. Joint Bone Spine 79: 591-596. S1297-319X(12)00024-3 [pii];10.1016/j.jbspin.2012.02.003 [doi].
