# Supplementary material for: Anti-TNF Treatment Response in Rheumatoid Arthritis Patients Is Associated with Genetic Variation in the NLRP3-Inflammasome
Source: PLoS One. 2014 Jun 26;9(6):e100361. doi: 10.1371/journal.pone.0100361 (PMC4072633; doi:10.1371/journal.pone.0100361)
Supplement: Table S2 — Adjusted odds ratios for associations between gene variants and EULAR anti-TNF treatment response. (a. All RA patients, b. Seropositive RA patients). (DOCX) [file pone.0100361.s003.docx]

| **Supplementary Table 2a.** RA patients - adjusted odds ratios for associations between gene variants and EULAR anti-TNF treatment response. | | | | | | | | | | | | | | | | | | |  |
| --- | --- | --- | --- | --- | --- | --- | --- | --- | --- | --- | --- | --- | --- | --- | --- | --- | --- | --- | --- |
|  |  |  |  |  |  | GOOD/MODERATE | | |  | GOOD | |  | |  | GOOD/MODERATE | | |  | |
| Gene  (SNP) | Genotype | Freq. | None | Moderate | Good | Adj. OR | 95% CI | P-Value | | Adj. OR | 95% CI | | P-Value | | Crude  OR | 95% CI | P-Value | |  |
| *CD14* | GG | 153 | 49 | 34 | 70 |  |  |  | |  |  | |  | |  |  |  | |  |
| rs2569190 | GA | 255 | 67 | 84 | 104 | 1.35 | (0.86-2.11) | 0.188 | | 1.12 | (0.69-1.83) | | 0.650 | |  |  |  | |  |
|  | AA | 94 | 32 | 21 | 41 | 0.94 | (0.54-1.63) | 0.821 | | 0.95 | (0.52-1.73) | | 0.856 | |  |  |  | |  |
|  | GA/AA | 349 | 99 | 105 | 145 | 1.22 | (0.80-1.85) | 0.358 | | 1.06 | (0.67-1.69) | | 0.791 | | 1.12 | (0.75-1.67) | 0.586 | |  |
| *IFNG* | TT | 137 | 34 | 37 | 66 |  |  |  | |  |  | |  | |  |  |  | |  |
| rs2430561 | TA | 263 | 74 | 71 | 118 | 0.81 | (0.50-1.31) | 0.395 | | 0.75 | (0.45-1.27) | | 0.285 | |  |  |  | |  |
|  | AA | 114 | 40 | 38 | 36 | 0.59 | (0.34-1.02) | 0.059 | | 0.40 | (0.21-0.76) | | 0.005** | |  |  |  | |  |
|  | TA/AA | 377 | 114 | 109 | 154 | 0.73 | (0.47-1.15) | 0.177 | | 0.63 | (0.38-1.03) | | 0.067 | | 0.79 | (0.51-1.22) | 0.290 | |  |
| *IL1B* | GG | 255 | 77 | 82 | 96 |  |  |  | |  |  | |  | |  |  |  | |  |
| rs1143623 | GC | 223 | 63 | 55 | 105 | 1.11 | (0.75-1.66) | 0.605 | | 1.33 | (0.85-2.07) | | 0.208 | |  |  |  | |  |
|  | CC | 37 | 11 | 9 | 17 | 1.06 | (0.50-2.27) | 0.878 | | 1.32 | (0.57-3.06) | | 0.510 | |  |  |  | |  |
|  | GC/CC | 260 | 74 | 64 | 122 | 1.10 | (0.75-1.62) | 0.614 | | 1.33 | (0.87-2.03) | | 0.192 | | 1.08 | (0.74-1.57) | 0.691 | |  |
| *IL1B* | TT | 209 | 59 | 71 | 79 |  |  |  | |  |  | |  | |  |  |  | |  |
| rs1143627 | TC | 239 | 74 | 58 | 107 | 0.88 | (0.59-1.33) | 0.556 | | 1.05 | (0.66-1.66) | | 0.836 | |  |  |  | |  |
|  | CC | 64 | 19 | 15 | 30 | 0.94 | (0.50-1.75) | 0.841 | | 1.25 | (0.63-2.51) | | 0.523 | |  |  |  | |  |
|  | TC/CC | 303 | 93 | 73 | 137 | 0.89 | (0.61-1.32) | 0.578 | | 1.09 | (0.70-1.69) | | 0.707 | | 0.90 | (0.62-1.32) | 0.587 | |  |
| *IL1B* | GG | 165 | 52 | 37 | 76 |  |  |  | |  |  | |  | |  |  |  | |  |
| rs4848306 | GA | 257 | 72 | 76 | 109 | 1.22 | (0.79-1.88) | 0.365 | | 1.04 | (0.65-1.67) | | 0.879 | |  |  |  | |  |
|  | AA | 91 | 26 | 32 | 33 | 1.15 | (0.65-2.04) | 0.619 | | 0.88 | (0.47-1.67) | | 0.702 | |  |  |  | |  |
|  | GA/AA | 348 | 98 | 108 | 142 | 1.20 | (0.80-1.81) | 0.374 | | 1.00 | (0.64-1.56) | | 0.989 | | 1.14 | (0.77-1.70) | 0.508 | |  |
| *IL1RN* | TT | 200 | 59 | 57 | 84 |  |  |  | |  |  | |  | |  |  |  | |  |
| rs4251961 | TC | 230 | 62 | 62 | 106 | 1.12 | (0.73-1.71) | 0.614 | | 1.21 | (0.76-1.93) | | 0.423 | |  |  |  | |  |
|  | CC | 80 | 28 | 24 | 28 | 0.74 | (0.42-1.30) | 0.301 | | 0.73 | (0.39-1.37) | | 0.323 | |  |  |  | |  |
|  | TC/CC | 310 | 90 | 86 | 134 | 1.00 | (0.67-1.49) | 0.993 | | 1.06 | (0.68-1.64) | | 0.791 | | 1.00 | (0.68-1.48) | 0.986 | |  |
| *IL4R* | AA | 169 | 52 | 43 | 74 |  |  |  | |  |  | |  | |  |  |  | |  |
| rs1805010 | AG | 264 | 76 | 79 | 109 | 1.08 | (0.70-1.67) | 0.725 | | 0.96 | (0.59-1.55) | | 0.862 | |  |  |  | |  |
|  | GG | 86 | 25 | 24 | 37 | 1.08 | (0.61-1.91) | 0.805 | | 0.97 | (0.52-1.84) | | 0.937 | |  |  |  | |  |
|  | AG/GG | 350 | 101 | 103 | 146 | 1.08 | (0.72-1.63) | 0.715 | | 0.96 | (0.61-1.51) | | 0.869 | | 1.12 | (0.75-1.65) | 0.588 | |  |
| *IL6* | TT | 318 | 97 | 96 | 125 |  |  |  | |  |  | |  | |  |  |  | |  |
| rs10499563 | TC | 177 | 48 | 45 | 84 | 1.17 | (0.77-1.77) | 0.460 | | 1.26 | (0.80-1.99) | | 0.325 | |  |  |  | |  |
|  | CC | 22 | 7 | 4 | 11 | 1.02 | (0.40-2.60) | 0.975 | | 1.28 | (0.47-3.51) | | 0.626 | |  |  |  | |  |
|  | TC/CC | 199 | 55 | 49 | 95 | 1.15 | (0.77-1.71) | 0.491 | | 1.26 | (0.81-1.96) | | 0.301 | | 1.13 | (0.77-1.66) | 0.532 | |  |
| *IL6R* | CC | 188 | 54 | 46 | 88 |  |  |  | |  |  | |  | |  |  |  | |  |
| rs4537545 | CT | 250 | 77 | 76 | 97 | 0.94 | (0.62-1.44) | 0.786 | | 0.81 | (0.51-1.28) | | 0.362 | |  |  |  | |  |
|  | TT | 76 | 20 | 23 | 33 | 1.20 | (0.65-2.21) | 0.559 | | 1.06 | (0.54-2.07) | | 0.867 | |  |  |  | |  |
|  | CT/TT | 326 | 97 | 99 | 130 | 1.00 | (0.67-1.49) | 0.982 | | 0.86 | (0.55-1.33) | | 0.492 | | 0.89 | (0.60-1.32) | 0.561 | |  |
| *IL10* | CC | 312 | 96 | 86 | 130 |  |  |  | |  |  | |  | |  |  |  | |  |
| rs1800872 | CA | 177 | 47 | 51 | 79 | 1.24 | (0.82-1.88) | 0.312 | | 1.23 | (0.78-1.94) | | 0.377 | |  |  |  | |  |
|  | AA | 26 | 7 | 9 | 10 | 1.19 | (0.48-2.97) | 0.710 | | 1.01 | (0.36-2.81) | | 0.992 | |  |  |  | |  |
|  | CA/AA | 203 | 54 | 60 | 89 | 1.23 | (0.83-1.84) | 0.303 | | 1.20 | (0.77-1.86) | | 0.416 | | 1.23 | (0.83-1.81) | 0.301 | |  |
| *IL10* | CC | 350 | 102 | 95 | 153 |  |  |  | |  |  | |  | |  |  |  | |  |
| rs3024505 | CT | 156 | 43 | 51 | 62 | 1.05 | (0.68-1.60) | 0.828 | | 0.91 | (0.57-1.47) | | 0.711 | |  |  |  | |  |
|  | TT | 12 | 6 | 0 | 6 | 0.39 | (0.12-1.26) | 0.117 | | 0.61 | (0.19-2.04) | | 0.427 | |  |  |  | |  |
|  | CT/TT | 168 | 49 | 51 | 68 | 0.97 | (0.64-1.46) | 0.879 | | 0.88 | (0.56-1.39) | | 0.576 | | 1.02 | (0.68-1.51) | 0.943 | |  |
| *IL17A* | GG | 234 | 62 | 70 | 102 |  |  |  | |  |  | |  | |  |  |  | |  |
| rs2275913 | GA | 228 | 73 | 61 | 94 | 0.79 | (0.53-1.19) | 0.260 | | 0.82 | (0.53-1.29) | | 0.399 | |  |  |  | |  |
|  | AA | 55 | 18 | 13 | 24 | 0.76 | (0.40-1.44) | 0.397 | | 0.81 | (0.40-1.64) | | 0.564 | |  |  |  | |  |
|  | GA/AA | 283 | 91 | 74 | 118 | 0.78 | (0.53-1.16) | 0.220 | | 0.82 | (0.54-1.26) | | 0.368 | | 0.77 | (0.53-1.12) | 0.163 | |  |
| *IL23R* | GG | 454 | 134 | 126 | 194 |  |  |  | |  |  | |  | |  |  |  | |  |
| rs11209026 | GA | 61 | 17 | 20 | 24 | 1.13 | (0.62-2.06) | 0.691 | | 1.05 | (0.54-2.07) | | 0.879 | |  |  |  | |  |
|  | AA | 1 | 0 | 0 | 1 | - | - | - | | - | - | | - | |  |  |  | |  |
|  | GA/AA | 62 | 17 | 20 | 25 | 1.15 | (0.63-2.10) | 0.641 | | 1.10 | (0.56-2.15) | | 0.778 | | 0.99 | (0.56-1.75) | 0.966 | |  |
| *LY96* | CC | 248 | 73 | 75 | 100 |  |  |  | |  |  | |  | |  |  |  | |  |
| rs11465996 | CG | 213 | 58 | 59 | 96 | 1.14 | (0.75-1.72) | 0.541 | | 1.29 | (0.82-2.04) | | 0.269 | |  |  |  | |  |
|  | GG | 54 | 19 | 12 | 23 | 0.78 | (0.42-1.46) | 0.435 | | 0.85 | (0.43-1.70) | | 0.647 | |  |  |  | |  |
|  | CG/GG | 267 | 77 | 71 | 119 | 1.05 | (0.71-1.54) | 0.813 | | 1.18 | (0.77-1.80) | | 0.453 | | 1.03 | (0.71-1.50) | 0.859 | |  |
| *MAP3K14* | TT | 151 | 42 | 43 | 66 |  |  |  | |  |  | |  | |  |  |  | |  |
| rs7222094 | TC | 254 | 69 | 84 | 101 | 1.11 | (0.70-1.75) | 0.664 | | 1.05 | (0.63-1.74) | | 0.860 | |  |  |  | |  |
|  | CC | 106 | 40 | 16 | 50 | 0.66 | (0.39-1.13) | 0.130 | | 0.88 | (0.49-1.57) | | 0.660 | |  |  |  | |  |
|  | TC/CC | 360 | 109 | 100 | 151 | 0.94 | (0.61-1.44) | 0.780 | | 0.98 | (0.61-1.58) | | 0.940 | | 0.87 | (0.58-1.32) | 0.524 | |  |
| *NFKB1* | ins/ins | 213 | 61 | 52 | 100 |  |  |  | |  |  | |  | |  |  |  | |  |
| rs28362491 | ins/- | 222 | 69 | 64 | 89 | 0.86 | (0.57-1.31) | 0.490 | | 0.77 | (0.49-1.22) | | 0.264 | |  |  |  | |  |
|  | -/- | 78 | 20 | 28 | 30 | 1.13 | (0.63-2.05) | 0.681 | | 0.86 | (0.44-1.67) | | 0.650 | |  |  |  | |  |
|  | ins/- or  -/- | 300 | 89 | 92 | 119 | 0.92 | (0.62-1.37) | 0.695 | | 0.79 | (0.51-1.22) | | 0.284 | | 0.96 | (0.66-1.41) | 0.840 | |  |
| *NFKBIA* | TT | 516 | 151 | 145 | 220 |  |  |  | |  |  | |  | |  |  |  | |  |
| rs17103265 | T/- | 2 | 0 | 1 | 1 | - | - | - | | - | - | | - | |  |  |  | |  |
|  | -/- | 0 | 0 | 0 | 0 | - | - | - | | - | - | | - | |  |  |  | |  |
|  | T/- or  -/- | 2 | 0 | 1 | 1 | - | - | - | | - | - | | - | | - | - | - | |  |
| *NFKBIA* | GG | 210 | 64 | 59 | 87 |  |  |  | |  |  | |  | |  |  |  | |  |
| rs696 | GA | 233 | 68 | 61 | 104 | 1.10 | (0.73-1.66) | 0.657 | | 1.22 | (0.78-1.93) | | 0.384 | |  |  |  | |  |
|  | AA | 67 | 16 | 23 | 28 | 1.55 | (0.81-2.96) | 0.182 | | 1.48 | (0.72-3.03) | | 0.287 | |  |  |  | |  |
|  | GA/AA | 300 | 84 | 84 | 132 | 1.18 | (0.80-1.75) | 0.406 | | 1.27 | (0.82-1.96) | | 0.282 | | 1.09 | (0.74-1.60) | 0.664 | |  |
| *NLRP3* | CC | 275 | 69 | 84 | 122 |  |  |  | |  |  | |  | |  |  |  | |  |
| rs4612666 | CT | 210 | 73 | 54 | 83 | 0.62 | (0.42-0.92) | 0.018* | | 0.62 | (0.40-0.97) | | 0.037* | |  |  |  | |  |
|  | TT | 31 | 9 | 8 | 14 | 0.85 | (0.37-1.96) | 0.707 | | 0.89 | (0.36-2.24) | | 0.808 | |  |  |  | |  |
|  | CT/TT | 241 | 82 | 62 | 97 | 0.64 | (0.44-0.95) | 0.025* | | 0.65 | (0.43-1.00) | | 0.050* | | 0.68 | (0.46-0.99) | 0.041 | |  |
| *PPARG* | CC | 403 | 118 | 113 | 172 |  |  |  | |  |  | |  | |  |  |  | |  |
| rs1801282 | CG | 100 | 31 | 29 | 40 | 0.93 | (0.57-1.50) | 0.762 | | 0.86 | (0.50-1.48) | | 0.587 | |  |  |  | |  |
|  | GG | 9 | 2 | 4 | 3 | 1.74 | (0.35-8.66) | 0.497 | | 1.29 | (0.20-8.22) | | 0.786 | |  |  |  | |  |
|  | CG/GG | 109 | 33 | 33 | 43 | 0.97 | (0.61-1.56) | 0.915 | | 0.89 | (0.52-1.50) | | 0.650 | | 0.92 | (0.59-1.44) | 0.718 | |  |
| *PTPN22* | GG | 366 | 104 | 112 | 150 |  |  |  | |  |  | |  | |  |  |  | |  |
| rs2476601 | GA | 138 | 40 | 30 | 68 | 0.98 | (0.63-1.51) | 0.912 | | 1.20 | (0.74-1.93) | | 0.453 | |  |  |  | |  |
|  | AA | 10 | 2 | 4 | 4 | 1.57 | (0.32-7.81) | 0.580 | | 2.07 | (0.35-12.40) | | 0.425 | |  |  |  | |  |
|  | GA/AA | 148 | 42 | 34 | 72 | 1.00 | (0.65-1.54) | 0.992 | | 1.23 | (0.77-1.97) | | 0.382 | | 1.01 | (0.67-1.54) | 0.948 | |  |
| *SUMO4* | TT | 160 | 43 | 46 | 71 |  |  |  | |  |  | |  | |  |  |  | |  |
| rs237025 | TC | 240 | 75 | 62 | 103 | 0.81 | (0.52-1.28) | 0.369 | | 0.80 | (0.49-1.31) | | 0.378 | |  |  |  | |  |
|  | CC | 117 | 33 | 38 | 46 | 0.96 | (0.56-1.64) | 0.875 | | 0.93 | (0.51-1.71) | | 0.826 | |  |  |  | |  |
|  | TC/CC | 357 | 108 | 100 | 149 | 0.86 | (0.56-1.31) | 0.476 | | 0.84 | (0.53-1.34) | | 0.462 | | 0.92 | (0.61-1.37) | 0.667 | |  |
| *TGFB1* | CC | 246 | 73 | 71 | 102 |  |  |  | |  |  | |  | |  |  |  | |  |
| rs1800469 | CT | 231 | 64 | 67 | 100 | 1.09 | (0.73-1.63) | 0.680 | | 1.09 | (0.70-1.70) | | 0.707 | |  |  |  | |  |
|  | TT | 42 | 15 | 8 | 19 | 0.74 | (0.37-1.48) | 0.388 | | 0.86 | (0.40-1.84) | | 0.703 | |  |  |  | |  |
|  | CT/TT | 273 | 79 | 75 | 119 | 1.02 | (0.70-1.50) | 0.914 | | 1.05 | (0.69-1.60) | | 0.836 | | 1.04 | (0.71-1.50) | 0.848 | |  |
| *TLR2* | CC | 231 | 69 | 64 | 98 |  |  |  | |  |  | |  | |  |  |  | |  |
| rs11938228 | CA | 232 | 68 | 66 | 98 | 1.01 | (0.67-1.51) | 0.981 | | 1.01 | (0.65-1.58) | | 0.965 | |  |  |  | |  |
|  | AA | 56 | 14 | 16 | 26 | 1.37 | (0.70-2.70) | 0.358 | | 1.40 | (0.67-2.92) | | 0.372 | |  |  |  | |  |
|  | CA/AA | 288 | 82 | 82 | 124 | 1.07 | (0.73-1.57) | 0.740 | | 1.08 | (0.70-1.64) | | 0.738 | | 1.12 | (0.77-1.62) | 0.568 | |  |
| *TLR2* | CC | 393 | 113 | 108 | 172 |  |  |  | |  |  | |  | |  |  |  | |  |
| rs1816702 | CT | 112 | 35 | 33 | 44 | 0.89 | (0.56-1.42) | 0.632 | | 0.80 | (0.48-1.34) | | 0.400 | |  |  |  | |  |
|  | TT | 0 | 0 | 0 | 0 | - | - | - | | - | - | | - | |  |  |  | |  |
|  | CT/TT | 112 | 35 | 33 | 44 | 0.89 | (0.56-1.42) | 0.632 | | 0.80 | (0.48-1.34) | | 0.400 | | 0.88 | (0.56-1.38) | 0.583 | |  |
| *TLR2* | TT | 167 | 53 | 41 | 73 |  |  |  | |  |  | |  | |  |  |  | |  |
| rs3804099 | TC | 250 | 76 | 68 | 106 | 1.03 | (0.67-1.59) | 0.877 | | 0.94 | (0.58-1.50) | | 0.787 | |  |  |  | |  |
|  | CC | 93 | 21 | 33 | 39 | 1.56 | (0.86-2.82) | 0.142 | | 1.30 | (0.68-2.50) | | 0.430 | |  |  |  | |  |
|  | TC/CC | 343 | 97 | 101 | 145 | 1.15 | (0.76-1.72) | 0.510 | | 1.01 | (0.65-1.59) | | 0.952 | | 1.09 | (0.73-1.62) | 0.672 | |  |
| *TLR2* | AA | 136 | 41 | 38 | 57 |  |  |  | |  |  | |  | |  |  |  | |  |
| rs4696480 | AT | 264 | 77 | 72 | 115 | 1.00 | (0.63-1.58) | 0.996 | | 1.00 | (0.60-1.67) | | 0.998 | |  |  |  | |  |
|  | TT | 116 | 33 | 36 | 47 | 1.11 | (0.64-1.93) | 0.705 | | 1.02 | (0.55-1.88) | | 0.958 | |  |  |  | |  |
|  | AT/TT | 380 | 110 | 108 | 162 | 1.03 | (0.67-1.59) | 0.878 | | 1.01 | (0.62-1.63) | | 0.982 | | 1.12 | (0.74-1.70) | 0.581 | |  |
| *TLR4* | TT | 189 | 52 | 49 | 88 |  |  |  | |  |  | |  | |  |  |  | |  |
| rs12377632 | TC | 243 | 75 | 71 | 97 | 0.84 | (0.55-1.28) | 0.417 | | 0.79 | (0.50-1.27) | | 0.338 | |  |  |  | |  |
|  | CC | 75 | 23 | 20 | 32 | 0.87 | (0.48-1.59) | 0.656 | | 0.80 | (0.41-1.53) | | 0.493 | |  |  |  | |  |
|  | TC/CC | 318 | 98 | 91 | 129 | 0.85 | (0.56-1.27) | 0.419 | | 0.79 | (0.51-1.24) | | 0.312 | | 0.89 | (0.60-1.32) | 0.562 | |  |
| *TLR4* | TT | 286 | 87 | 83 | 116 |  |  |  | |  |  | |  | |  |  |  | |  |
| rs1554973 | TC | 201 | 55 | 56 | 90 | 1.15 | (0.77-1.72) | 0.505 | | 1.22 | (0.78-1.91) | | 0.376 | |  |  |  | |  |
|  | CC | 30 | 10 | 5 | 15 | 0.86 | (0.38-1.92) | 0.707 | | 1.10 | (0.46-2.60) | | 0.829 | |  |  |  | |  |
|  | TC/CC | 231 | 65 | 61 | 105 | 1.10 | (0.75-1.62) | 0.620 | | 1.20 | (0.79-1.84) | | 0.393 | | 1.12 | (0.77-1.63) | 0.549 | |  |
| *TLR4* | GG | 253 | 76 | 68 | 109 |  |  |  | |  |  | |  | |  |  |  | |  |
| rs5030728 | GA | 215 | 60 | 60 | 95 | 1.09 | (0.73-1.65) | 0.667 | | 1.13 | (0.72-1.76) | | 0.606 | |  |  |  | |  |
|  | AA | 46 | 14 | 17 | 15 | 1.01 | (0.51-2.00) | 0.985 | | 0.75 | (0.34-1.67) | | 0.481 | |  |  |  | |  |
|  | GA/AA | 261 | 74 | 77 | 110 | 1.08 | (0.73-1.58) | 0.706 | | 1.05 | (0.69-1.61) | | 0.813 | | 1.05 | (0.72-1.52) | 0.811 | |  |
| *TLR5* | CC | 448 | 129 | 129 | 190 |  |  |  | |  |  | |  | |  |  |  | |  |
| rs5744168 | CT | 66 | 20 | 17 | 29 | 0.91 | (0.51-1.6) | 0.740 | | 0.96 | (0.52-1.80) | | 0.906 | |  |  |  | |  |
|  | TT | 2 | 1 | 0 | 1 | 0.37 | (0.02-6.00) | 0.482 | | 0.62 | (0.04-10.29) | | 0.739 | |  |  |  | |  |
|  | CT/TT | 68 | 21 | 17 | 30 | 0.88 | (0.50-1.54) | 0.659 | | 0.95 | (0.51-1.74) | | 0.859 | | 0.91 | (0.53-1.56) | 0.733 | |  |
| *TLR9* | TT | 188 | 51 | 51 | 86 |  |  |  | |  |  | |  | |  |  |  | |  |
| rs187084 | TC | 243 | 71 | 68 | 104 | 0.93 | (0.61-1.43) | 0.739 | | 0.90 | (0.56-1.43) | | 0.648 | |  |  |  | |  |
|  | CC | 87 | 29 | 26 | 32 | 0.76 | (0.44-1.33) | 0.343 | | 0.69 | (0.37-1.29) | | 0.250 | |  |  |  | |  |
|  | TC/CC | 330 | 100 | 94 | 136 | 0.88 | (0.59-1.32) | 0.540 | | 0.84 | (0.54-1.30) | | 0.431 | | 0.84 | (0.56-1.24) | 0.376 | |  |
| *TLR9* | GG | 165 | 53 | 47 | 65 |  |  |  | |  |  | |  | |  |  |  | |  |
| rs352139 | GA | 233 | 70 | 64 | 99 | 1.10 | (0.71-1.70) | 0.675 | | 1.11 | (0.68-1.82) | | 0.662 | |  |  |  | |  |
|  | AA | 113 | 28 | 33 | 52 | 1.44 | (0.83-2.48) | 0.191 | | 1.46 | (0.80-2.65) | | 0.216 | |  |  |  | |  |
|  | GA/AA | 346 | 98 | 97 | 151 | 1.19 | (0.80-1.80) | 0.392 | | 1.21 | (0.77-1.91) | | 0.402 | | 1.15 | (0.78-1.71) | 0.479 | |  |
| *TNF* | GG | 348 | 100 | 96 | 152 |  |  |  | |  |  | |  | |  |  |  | |  |
| rs1800629 | GA | 155 | 45 | 46 | 64 | 0.97 | (0.64-1.48) | 0.894 | | 0.94 | (0.59-1.50) | | 0.797 | |  |  |  | |  |
|  | AA | 12 | 4 | 3 | 5 | 0.81 | (0.23-2.83) | 0.740 | | 0.99 | (0.24-4.07) | | 0.984 | |  |  |  | |  |
|  | GA/AA | 167 | 49 | 49 | 69 | 0.96 | (0.63-1.45) | 0.841 | | 0.94 | (0.60-1.49) | | 0.803 | | 0.98 | (0.65-1.45) | 0.904 | |  |
| *TNF* | GG | 493 | 145 | 138 | 210 |  |  |  | |  |  | |  | |  |  |  | |  |
| rs361525 | GA | 23 | 5 | 7 | 11 | 1.64 | (0.59-4.54) | 0.343 | | 1.71 | (0.57-5.16) | | 0.339 | |  |  |  | |  |
|  | AA | 1 | 0 | 0 | 1 | - | - | - | | - | - | | - | |  |  |  | |  |
|  | GA/AA | 24 | 5 | 7 | 12 | 1.74 | (0.63-4.79) | 0.284 | | 1.91 | (0.64-5.65) | | 0.245 | | 1.60 | (0.59-4.37) | 0.356 | |  |
| *TNFAIP3* | CC | 298 | 85 | 82 | 131 |  |  |  | |  |  | |  | |  |  |  | |  |
| rs6927172 | CG | 187 | 59 | 50 | 78 | 0.88 | (0.59-1.31) | 0.523 | | 0.89 | (0.57-1.39) | | 0.615 | |  |  |  | |  |
|  | GG | 31 | 7 | 13 | 11 | 1.39 | (0.57-3.38) | 0.463 | | 1.03 | (0.38-2.83) | | 0.954 | |  |  |  | |  |
|  | CG/GG | 218 | 66 | 63 | 89 | 0.93 | (0.63-1.37) | 0.723 | | 0.91 | (0.59-1.39) | | 0.655 | | 0.89 | (0.61-1.30) | 0.537 | |  |
| *TNFRSF1A* | TT | 180 | 56 | 50 | 74 |  |  |  | |  |  | |  | |  |  |  | |  |
| rs1800693 | TC | 250 | 72 | 71 | 107 | 1.10 | (0.72-1.68) | 0.665 | | 1.07 | (0.67-1.71) | | 0.779 | |  |  |  | |  |
|  | CC | 88 | 24 | 25 | 39 | 1.14 | (0.65-2.03) | 0.643 | | 1.13 | (0.60-2.12) | | 0.710 | |  |  |  | |  |
|  | TC/CC | 338 | 96 | 96 | 146 | 1.11 | (0.74-1.66) | 0.610 | | 1.08 | (0.70-1.69) | | 0.722 | | 1.18 | (0.80-1.74) | 0.390 | |  |
| *TNFRSF1A* | GG | 178 | 46 | 54 | 78 |  |  |  | |  |  | |  | |  |  |  | |  |
| rs4149570 | GT | 261 | 85 | 68 | 108 | 0.75 | (0.49-1.15) | 0.182 | | 0.78 | (0.49-1.26) | | 0.314 | |  |  |  | |  |
|  | TT | 76 | 22 | 23 | 31 | 0.92 | (0.50-1.69) | 0.778 | | 0.91 | (0.46-1.78) | | 0.776 | |  |  |  | |  |
|  | GT/TT | 337 | 107 | 91 | 139 | 0.78 | (0.52-1.18) | 0.237 | | 0.81 | (0.51-1.27) | | 0.359 | | 0.74 | (0.49-1.10) | 0.137 | |  |
| *TNFRSF10A* | GG | 134 | 42 | 34 | 58 |  |  |  | |  |  | |  | |  |  |  | |  |
| rs20575 | GC | 249 | 78 | 67 | 104 | 1.02 | (0.64-1.61) | 0.942 | | 1.00 | (0.60-1.65) | | 0.991 | |  |  |  | |  |
|  | CC | 132 | 32 | 45 | 55 | 1.44 | (0.83-2.49) | 0.192 | | 1.31 | (0.72-2.40) | | 0.374 | |  |  |  | |  |
|  | GC/CC | 381 | 110 | 112 | 159 | 1.14 | (0.74-1.75) | 0.555 | | 1.09 | (0.68-1.75) | | 0.725 | | 1.08 | (0.71-1.65) | 0.711 | |  |
| Logistic regression, adjusted for gender, age, HAQ-, DMARD at baseline, CRP, RA diagnosis (seropositive/seronegative). Adj. OR: adjusted odds ratio; Crude OR under assumption of a dominant genetic model | | | | | | | | | | | | | | | | | | |  |

| **Supplementary Table 2b.** Seropositive RA patients - adjusted odds ratios for associations between gene variants and EULAR anti-TNF treatment response. | | | | | | | | | | | | | | | | | | | |
| --- | --- | --- | --- | --- | --- | --- | --- | --- | --- | --- | --- | --- | --- | --- | --- | --- | --- | --- | --- |
|  |  |  |  |  |  | GOOD/MODERATE | | |  | GOOD | |  |  | | GOOD/MODERATE | | | |  |
| Gene  (SNP) | Genotype | Freq. | None | Moderate | Good | Adj. OR | 95% CI | P-Value | | Adj. OR | 95% CI | | | P-Value | | Crude  OR | 95% CI | P-Value | |
| *CD14* | GG | 116 | 37 | 24 | 55 |  |  |  | |  |  | | |  | |  |  |  | |
| rs2569190 | GA | 193 | 51 | 62 | 80 | 1.32 | (0.79-2.2) | 0.289 | | 1.12 | (0.64-1.96) | | | 0.703 | |  |  |  | |
|  | AA | 71 | 24 | 15 | 32 | 0.92 | (0.49-1.75) | 0.800 | | 0.95 | (0.47-1.89) | | | 0.875 | |  |  |  | |
|  | GA/AA | 264 | 75 | 77 | 112 | 1.19 | (0.74-1.93) | 0.470 | | 1.06 | (0.63-1.79) | | | 0.825 | | 1.14 | (0.72-1.81) | 0.587 | |
| *IFNG* | TT | 106 | 26 | 30 | 50 |  |  |  | |  |  | | |  | |  |  |  | |
| rs2430561 | TA | 205 | 56 | 55 | 94 | 0.85 | (0.49-1.46) | 0.547 | | 0.83 | (0.46-1.51) | | | 0.544 | |  |  |  | |
|  | AA | 78 | 30 | 21 | 27 | 0.51 | (0.26-0.96) | 0.038* | | 0.42 | (0.2-0.87) | | | 0.020* | |  |  |  | |
|  | TA/AA | 283 | 86 | 76 | 121 | 0.73 | (0.43-1.22) | 0.229 | | 0.69 | (0.39-1.21) | | | 0.196 | | 0.78 | (0.47-1.27) | 0.314 | |
| *IL1B* | GG | 191 | 60 | 61 | 70 |  |  |  | |  |  | | |  | |  |  |  | |
| rs1143623 | GC | 169 | 45 | 39 | 85 | 1.26 | (0.79-2) | 0.326 | | 1.56 | (0.94-2.61) | | | 0.086 | |  |  |  | |
|  | CC | 30 | 10 | 6 | 14 | 0.92 | (0.4-2.09) | 0.838 | | 1.18 | (0.48-2.92) | | | 0.715 | |  |  |  | |
|  | GC/CC | 199 | 55 | 45 | 99 | 1.20 | (0.77-1.86) | 0.421 | | 1.50 | (0.92-2.44) | | | 0.106 | | 1.16 | (0.76-1.79) | 0.487 | |
| *IL1B* | TT | 158 | 47 | 54 | 57 |  |  |  | |  |  | | |  | |  |  |  | |
| rs1143627 | TC | 177 | 53 | 40 | 84 | 0.99 | (0.61-1.58) | 0.953 | | 1.26 | (0.74-2.15) | | | 0.387 | |  |  |  | |
|  | CC | 54 | 15 | 12 | 27 | 1.07 | (0.54-2.15) | 0.841 | | 1.47 | (0.68-3.17) | | | 0.327 | |  |  |  | |
|  | TC/CC | 231 | 68 | 52 | 111 | 1.01 | (0.64-1.57) | 0.981 | | 1.31 | (0.79-2.17) | | | 0.300 | | 1.01 | (0.65-1.56) | 0.961 | |
| *IL1B* | GG | 130 | 40 | 28 | 62 |  |  |  | |  |  | | |  | |  |  |  | |
| rs4848306 | GA | 198 | 54 | 57 | 87 | 1.25 | (0.76-2.05) | 0.382 | | 1.09 | (0.63-1.86) | | | 0.765 | |  |  |  | |
|  | AA | 63 | 21 | 21 | 21 | 0.88 | (0.46-1.69) | 0.711 | | 0.67 | (0.32-1.41) | | | 0.294 | |  |  |  | |
|  | GA/AA | 261 | 75 | 78 | 108 | 1.14 | (0.72-1.82) | 0.577 | | 0.97 | (0.58-1.61) | | | 0.902 | | 1.08 | (0.69-1.69) | 0.739 | |
| *IL1RN* | TT | 149 | 45 | 40 | 64 |  |  |  | |  |  | | |  | |  |  |  | |
| rs4251961 | TC | 178 | 47 | 47 | 84 | 1.17 | (0.72-1.91) | 0.522 | | 1.23 | (0.72-2.1) | | | 0.451 | |  |  |  | |
|  | CC | 58 | 20 | 16 | 22 | 0.78 | (0.4-1.51) | 0.459 | | 0.83 | (0.4-1.74) | | | 0.625 | |  |  |  | |
|  | TC/CC | 236 | 67 | 63 | 106 | 1.06 | (0.67-1.67) | 0.808 | | 1.11 | (0.67-1.84) | | | 0.672 | | 1.07 | (0.69-1.66) | 0.769 | |
| *IL4R* | AA | 126 | 37 | 30 | 59 |  |  |  | |  |  | | |  | |  |  |  | |
| rs1805010 | AG | 204 | 62 | 57 | 85 | 0.95 | (0.58-1.56) | 0.837 | | 0.86 | (0.5-1.48) | | | 0.575 | |  |  |  | |
|  | GG | 64 | 17 | 19 | 28 | 1.17 | (0.59-2.32) | 0.647 | | 1.00 | (0.48-2.12) | | | 0.990 | |  |  |  | |
|  | AG/GG | 268 | 79 | 76 | 113 | 1.00 | (0.62-1.61) | 0.999 | | 0.89 | (0.53-1.49) | | | 0.658 | | 1.03 | (0.65-1.63) | 0.897 | |
| *IL6* | TT | 243 | 74 | 70 | 99 |  |  |  | |  |  | | |  | |  |  |  | |
| rs10499563 | TC | 135 | 35 | 34 | 66 | 1.19 | (0.73-1.92) | 0.483 | | 1.27 | (0.75-2.15) | | | 0.377 | |  |  |  | |
|  | CC | 14 | 6 | 1 | 7 | 0.59 | (0.19-1.78) | 0.346 | | 0.85 | (0.27-2.71) | | | 0.788 | |  |  |  | |
|  | TC/CC | 149 | 41 | 35 | 73 | 1.10 | (0.7-1.75) | 0.678 | | 1.21 | (0.73-2) | | | 0.462 | | 1.16 | (0.74-1.81) | 0.517 | |
| *IL6R* | CC | 137 | 40 | 32 | 65 |  |  |  | |  |  | | |  | |  |  |  | |
| rs4537545 | CT | 192 | 58 | 57 | 77 | 1.01 | (0.62-1.63) | 0.983 | | 0.87 | (0.51-1.48) | | | 0.605 | |  |  |  | |
|  | TT | 59 | 16 | 16 | 27 | 1.21 | (0.6-2.42) | 0.599 | | 1.12 | (0.52-2.39) | | | 0.780 | |  |  |  | |
|  | CT/TT | 251 | 74 | 73 | 104 | 1.05 | (0.66-1.67) | 0.847 | | 0.92 | (0.55-1.53) | | | 0.744 | | 0.92 | (0.59-1.45) | 0.725 | |
| *IL10* | CC | 236 | 68 | 67 | 101 |  |  |  | |  |  | | |  | |  |  |  | |
| rs1800872 | CA | 132 | 40 | 32 | 60 | 0.92 | (0.57-1.48) | 0.740 | | 0.99 | (0.59-1.67) | | | 0.983 | |  |  |  | |
|  | AA | 21 | 5 | 7 | 9 | 1.36 | (0.47-3.96) | 0.567 | | 1.23 | (0.38-3.99) | | | 0.725 | |  |  |  | |
|  | CA/AA | 153 | 45 | 39 | 69 | 0.97 | (0.62-1.53) | 0.898 | | 1.02 | (0.62-1.68) | | | 0.938 | | 0.96 | (0.62-1.49) | 0.860 | |
| *IL10* | CC | 266 | 78 | 67 | 121 |  |  |  | |  |  | | |  | |  |  |  | |
| rs3024505 | CT | 119 | 33 | 39 | 47 | 1.04 | (0.64-1.7) | 0.864 | | 0.87 | (0.51-1.5) | | | 0.620 | |  |  |  | |
|  | TT | 9 | 5 | 0 | 4 | 0.30 | (0.08-1.16) | 0.081 | | 0.44 | (0.11-1.75) | | | 0.244 | |  |  |  | |
|  | CT/TT | 128 | 38 | 39 | 51 | 0.95 | (0.59-1.51) | 0.819 | | 0.81 | (0.48-1.37) | | | 0.437 | | 1.00 | (0.64-1.58) | 0.992 | |
| *IL17A* | GG | 176 | 51 | 50 | 75 |  |  |  | |  |  | | |  | |  |  |  | |
| rs2275913 | GA | 172 | 50 | 45 | 77 | 1.07 | (0.66-1.71) | 0.792 | | 1.17 | (0.69-1.97) | | | 0.555 | |  |  |  | |
|  | AA | 44 | 15 | 10 | 19 | 0.81 | (0.4-1.65) | 0.559 | | 0.91 | (0.42-1.98) | | | 0.815 | |  |  |  | |
|  | GA/AA | 216 | 65 | 55 | 96 | 1.00 | (0.64-1.57) | 0.987 | | 1.11 | (0.68-1.81) | | | 0.684 | | 0.98 | (0.64-1.5) | 0.912 | |
| *IL23R* | GG | 346 | 103 | 90 | 153 |  |  |  | |  |  | | |  | |  |  |  | |
| rs11209026 | GA | 43 | 11 | 16 | 16 | 1.35 | (0.65-2.81) | 0.423 | | 1.03 | (0.45-2.37) | | | 0.947 | |  |  |  | |
|  | AA | 1 | 0 | 0 | 1 | 0.00 | (0-0) | 0.000 | | 0.00 | (0-0) | | | 0.000 | |  |  |  | |
|  | GA/AA | 44 | 11 | 16 | 17 | 1.38 | (0.67-2.88) | 0.383 | | 1.09 | (0.48-2.49) | | | 0.836 | | 1.18 | (0.58-2.37) | 0.648 | |
| *LY96* | CC | 188 | 53 | 54 | 81 |  |  |  | |  |  | | |  | |  |  |  | |
| rs11465996 | CG | 162 | 47 | 43 | 72 | 0.96 | (0.6-1.54) | 0.875 | | 1.09 | (0.65-1.83) | | | 0.752 | |  |  |  | |
|  | GG | 41 | 14 | 9 | 18 | 0.76 | (0.37-1.58) | 0.462 | | 0.81 | (0.36-1.8) | | | 0.601 | |  |  |  | |
|  | CG/GG | 203 | 61 | 52 | 90 | 0.92 | (0.59-1.42) | 0.696 | | 1.02 | (0.63-1.66) | | | 0.940 | | 0.91 | (0.59-1.4) | 0.664 | |
| *MAP3K14* | TT | 117 | 34 | 30 | 53 |  |  |  | |  |  | | |  | |  |  |  | |
| rs7222094 | TC | 192 | 52 | 60 | 80 | 1.14 | (0.68-1.92) | 0.610 | | 1.04 | (0.59-1.84) | | | 0.890 | |  |  |  | |
|  | CC | 78 | 28 | 13 | 37 | 0.73 | (0.39-1.35) | 0.320 | | 0.86 | (0.44-1.69) | | | 0.670 | |  |  |  | |
|  | TC/CC | 270 | 80 | 73 | 117 | 0.99 | (0.61-1.61) | 0.980 | | 0.98 | (0.58-1.66) | | | 0.940 | | 0.98 | (0.61-1.56) | 0.927 | |
| *NFKB1* | ins/ins | 157 | 49 | 34 | 74 |  |  |  | |  |  | | |  | |  |  |  | |
| rs28362491 | ins/- | 176 | 51 | 51 | 74 | 1.10 | (0.69-1.77) | 0.690 | | 0.95 | (0.57-1.6) | | | 0.856 | |  |  |  | |
|  | -/- | 56 | 15 | 19 | 22 | 1.25 | (0.63-2.5) | 0.522 | | 0.93 | (0.43-2) | | | 0.846 | |  |  |  | |
|  | ins/- or  -/- | 232 | 66 | 70 | 96 | 1.14 | (0.73-1.77) | 0.577 | | 0.95 | (0.58-1.55) | | | 0.828 | | 1.12 | (0.72-1.72) | 0.622 | |
| *NFKBIA* | TT | 390 | 114 | 105 | 171 |  |  |  | |  |  | | |  | |  |  |  | |
| rs17103265 | T/- | 2 | 0 | 1 | 1 | - | - | - | | - | - | | | - | |  |  |  | |
|  | -/- |  | 0 |  |  | - | - | - | | - | - | | | - | |  |  |  | |
|  | T/- or  -/- | 2 | 0 | 1 | 1 | - | - | - | | - | - | | | - | | - | - | - | |
| *NFKBIA* | GG | 159 | 50 | 41 | 68 |  |  |  | |  |  | | |  | |  |  |  | |
| rs696 | GA | 179 | 52 | 48 | 79 | 1.14 | (0.71-1.83) | 0.578 | | 1.23 | (0.73-2.06) | | | 0.444 | |  |  |  | |
|  | AA | 49 | 11 | 14 | 24 | 1.67 | (0.79-3.56) | 0.182 | | 1.81 | (0.8-4.13) | | | 0.156 | |  |  |  | |
|  | GA/AA | 228 | 63 | 62 | 103 | 1.23 | (0.79-1.93) | 0.356 | | 1.33 | (0.81-2.18) | | | 0.264 | | 1.19 | (0.77-1.84) | 0.442 | |
| *NLRP3* | CC | 212 | 51 | 62 | 99 |  |  |  | |  |  | | |  | |  |  |  | |
| rs4612666 | CT | 156 | 55 | 39 | 62 | 0.58 | (0.37-0.92) | 0.020* | | 0.58 | (0.35-0.96) | | | 0.035* | |  |  |  | |
|  | TT | 25 | 9 | 5 | 11 | 0.59 | (0.24-1.43) | 0.241 | | 0.64 | (0.24-1.71) | | | 0.375 | |  |  |  | |
|  | CT/TT | 181 | 64 | 44 | 73 | 0.58 | (0.37-0.9) | 0.016* | | 0.59 | (0.36-0.96) | | | 0.032* | | 0.60 | (0.39-0.93) | 0.021* | |
| *PPARG* | CC | 307 | 91 | 80 | 136 |  |  |  | |  |  | | |  | |  |  |  | |
| rs1801282 | CG | 74 | 23 | 22 | 29 | 0.97 | (0.56-1.7) | 0.924 | | 0.81 | (0.43-1.53) | | | 0.518 | |  |  |  | |
|  | GG | 8 | 1 | 4 | 3 | 3.86 | (0.46-32.4) | 0.214 | | 3.04 | (0.3-30.7) | | | 0.346 | |  |  |  | |
|  | CG/GG | 82 | 24 | 26 | 32 | 1.08 | (0.63-1.87) | 0.774 | | 0.89 | (0.48-1.65) | | | 0.717 | | 0.96 | (0.57-1.6) | 0.861 | |
| *PTPN22* | GG | 269 | 77 | 78 | 114 |  |  |  | |  |  | | |  | |  |  |  | |
| rs2476601 | GA | 113 | 32 | 24 | 57 | 1.02 | (0.63-1.67) | 0.930 | | 1.23 | (0.72-2.09) | | | 0.454 | |  |  |  | |
|  | AA | 8 | 2 | 4 | 2 | 1.17 | (0.22-6.16) | 0.851 | | 0.79 | (0.1-6.08) | | | 0.820 | |  |  |  | |
|  | GA/AA | 121 | 34 | 28 | 59 | 1.03 | (0.64-1.67) | 0.902 | | 1.20 | (0.71-2.03) | | | 0.492 | | 1.05 | (0.65-1.67) | 0.853 | |
| *SUMO4* | TT | 124 | 33 | 33 | 58 |  |  |  | |  |  | | |  | |  |  |  | |
| rs237025 | TC | 173 | 53 | 45 | 75 | 0.82 | (0.49-1.38) | 0.466 | | 0.78 | (0.44-1.38) | | | 0.393 | |  |  |  | |
|  | CC | 94 | 28 | 28 | 38 | 0.88 | (0.48-1.61) | 0.683 | | 0.87 | (0.44-1.69) | | | 0.675 | |  |  |  | |
|  | TC/CC | 267 | 81 | 73 | 113 | 0.84 | (0.52-1.37) | 0.490 | | 0.81 | (0.48-1.37) | | | 0.430 | | 0.93 | (0.58-1.47) | 0.746 | |
| *TGFB1* | CC | 189 | 52 | 53 | 84 |  |  |  | |  |  | | |  | |  |  |  | |
| rs1800469 | CT | 174 | 54 | 48 | 72 | 0.85 | (0.54-1.34) | 0.480 | | 0.84 | (0.51-1.39) | | | 0.499 | |  |  |  | |
|  | TT | 31 | 10 | 5 | 16 | 0.79 | (0.34-1.8) | 0.571 | | 0.97 | (0.4-2.35) | | | 0.942 | |  |  |  | |
|  | CT/TT | 205 | 64 | 53 | 88 | 0.84 | (0.54-1.3) | 0.433 | | 0.86 | (0.53-1.39) | | | 0.542 | | 0.81 | (0.53-1.24) | 0.327 | |
| *TLR2* | CC | 175 | 52 | 45 | 78 |  |  |  | |  |  | | |  | |  |  |  | |
| rs11938228 | CA | 180 | 52 | 52 | 76 | 1.01 | (0.63-1.61) | 0.974 | | 0.91 | (0.55-1.52) | | | 0.721 | |  |  |  | |
|  | AA | 39 | 11 | 9 | 19 | 1.08 | (0.5-2.35) | 0.838 | | 1.15 | (0.5-2.67) | | | 0.738 | |  |  |  | |
|  | CA/AA | 219 | 63 | 61 | 95 | 1.02 | (0.66-1.59) | 0.925 | | 0.95 | (0.59-1.55) | | | 0.848 | | 1.11 | (0.72-1.71) | 0.630 | |
| *TLR2* | CC | 293 | 90 | 76 | 127 |  |  |  | |  |  | | |  | |  |  |  | |
| rs1816702 | CT | 87 | 22 | 25 | 40 | 1.30 | (0.75-2.26) | 0.352 | | 1.18 | (0.64-2.16) | | | 0.593 | |  |  |  | |
|  | TT |  | 0 |  |  | 0.00 | (0-0) | 0.000 | | 0.00 | (0-0) | | | 0.000 | |  |  |  | |
|  | CT/TT | 87 | 22 | 25 | 40 | 1.30 | (0.75-2.26) | 0.352 | | 1.18 | (0.64-2.16) | | | 0.593 | | 1.36 | (0.79-2.34) | 0.260 | |
| *TLR2* | TT | 121 | 37 | 29 | 55 |  |  |  | |  |  | | |  | |  |  |  | |
| rs3804099 | TC | 191 | 60 | 49 | 82 | 0.91 | (0.55-1.5) | 0.705 | | 0.84 | (0.49-1.46) | | | 0.543 | |  |  |  | |
|  | CC | 75 | 18 | 24 | 33 | 1.36 | (0.7-2.66) | 0.360 | | 1.24 | (0.6-2.57) | | | 0.568 | |  |  |  | |
|  | TC/CC | 266 | 78 | 73 | 115 | 1.01 | (0.63-1.63) | 0.965 | | 0.93 | (0.55-1.57) | | | 0.788 | | 0.98 | (0.62-1.56) | 0.945 | |
| *TLR2* | AA | 102 | 34 | 25 | 43 |  |  |  | |  |  | | |  | |  |  |  | |
| rs4696480 | AT | 202 | 56 | 56 | 90 | 1.22 | (0.72-2.06) | 0.465 | | 1.09 | (0.61-1.95) | | | 0.771 | |  |  |  | |
|  | TT | 89 | 25 | 25 | 39 | 1.23 | (0.66-2.3) | 0.517 | | 1.09 | (0.55-2.19) | | | 0.798 | |  |  |  | |
|  | AT/TT | 291 | 81 | 81 | 129 | 1.22 | (0.74-2) | 0.429 | | 1.09 | (0.63-1.89) | | | 0.754 | | 1.40 | (0.87-2.24) | 0.162 | |
| *TLR4* | TT | 147 | 38 | 38 | 71 |  |  |  | |  |  | | |  | |  |  |  | |
| rs12377632 | TC | 189 | 59 | 55 | 75 | 0.78 | (0.48-1.27) | 0.311 | | 0.70 | (0.41-1.2) | | | 0.195 | |  |  |  | |
|  | CC | 50 | 17 | 10 | 23 | 0.68 | (0.34-1.37) | 0.278 | | 0.69 | (0.32-1.49) | | | 0.347 | |  |  |  | |
|  | TC/CC | 239 | 76 | 65 | 98 | 0.75 | (0.47-1.2) | 0.237 | | 0.70 | (0.42-1.17) | | | 0.170 | | 0.81 | (0.52-1.27) | 0.364 | |
| *TLR4* | TT | 211 | 66 | 58 | 87 |  |  |  | |  |  | | |  | |  |  |  | |
| rs1554973 | TC | 156 | 40 | 43 | 73 | 1.31 | (0.82-2.09) | 0.260 | | 1.38 | (0.82-2.3) | | | 0.221 | |  |  |  | |
|  | CC | 25 | 9 | 4 | 12 | 0.77 | (0.32-1.85) | 0.560 | | 0.96 | (0.38-2.46) | | | 0.937 | |  |  |  | |
|  | TC/CC | 181 | 49 | 47 | 85 | 1.21 | (0.78-1.88) | 0.401 | | 1.30 | (0.8-2.12) | | | 0.290 | | 1.21 | (0.79-1.86) | 0.385 | |
| *TLR4* | GG | 186 | 57 | 44 | 85 |  |  |  | |  |  | | |  | |  |  |  | |
| rs5030728 | GA | 174 | 49 | 50 | 75 | 1.11 | (0.7-1.76) | 0.666 | | 1.05 | (0.63-1.74) | | | 0.851 | |  |  |  | |
|  | AA | 32 | 10 | 11 | 11 | 1.00 | (0.44-2.26) | 0.998 | | 0.72 | (0.28-1.86) | | | 0.502 | |  |  |  | |
|  | GA/AA | 206 | 59 | 61 | 86 | 1.09 | (0.7-1.69) | 0.706 | | 1.00 | (0.61-1.62) | | | 0.984 | | 1.06 | (0.69-1.62) | 0.806 | |
| *TLR5* | CC | 340 | 98 | 92 | 150 |  |  |  | |  |  | | |  | |  |  |  | |
| rs5744168 | CT | 50 | 15 | 14 | 21 | 0.91 | (0.47-1.75) | 0.772 | | 0.88 | (0.43-1.81) | | | 0.727 | |  |  |  | |
|  | TT | 2 | 1 | 0 | 1 | 0.36 | (0.02-5.8) | 0.469 | | 0.62 | (0.04-10.13) | | | 0.735 | |  |  |  | |
|  | CT/TT | 52 | 16 | 14 | 22 | 0.87 | (0.46-1.66) | 0.676 | | 0.86 | (0.43-1.75) | | | 0.681 | | 0.90 | (0.48-1.66) | 0.726 | |
| *TLR9* | TT | 137 | 34 | 35 | 68 |  |  |  | |  |  | | |  | |  |  |  | |
| rs187084 | TC | 189 | 59 | 49 | 81 | 0.75 | (0.46-1.24) | 0.265 | | 0.71 | (0.41-1.22) | | | 0.211 | |  |  |  | |
|  | CC | 67 | 22 | 21 | 24 | 0.71 | (0.37-1.35) | 0.290 | | 0.60 | (0.29-1.24) | | | 0.170 | |  |  |  | |
|  | TC/CC | 256 | 81 | 70 | 105 | 0.74 | (0.46-1.19) | 0.212 | | 0.68 | (0.41-1.14) | | | 0.140 | | 0.69 | (0.43-1.1) | 0.114 | |
| *TLR9* | GG | 126 | 42 | 34 | 50 |  |  |  | |  |  | | |  | |  |  |  | |
| rs352139 | GA | 182 | 52 | 51 | 79 | 1.26 | (0.76-2.06) | 0.369 | | 1.22 | (0.7-2.12) | | | 0.482 | |  |  |  | |
|  | AA | 81 | 20 | 21 | 40 | 1.53 | (0.81-2.88) | 0.185 | | 1.65 | (0.83-3.29) | | | 0.155 | |  |  |  | |
|  | GA/AA | 263 | 72 | 72 | 119 | 1.33 | (0.84-2.12) | 0.224 | | 1.34 | (0.8-2.24) | | | 0.267 | | 1.31 | (0.83-2.06) | 0.239 | |
| *TNF* | GG | 270 | 76 | 72 | 122 |  |  |  | |  |  | | |  | |  |  |  | |
| rs1800629 | GA | 116 | 36 | 31 | 49 | 0.86 | (0.53-1.38) | 0.528 | | 0.83 | (0.49-1.42) | | | 0.501 | |  |  |  | |
|  | AA | 5 | 2 | 2 | 1 | 0.56 | (0.09-3.5) | 0.531 | | 0.35 | (0.03-4.23) | | | 0.408 | |  |  |  | |
|  | GA/AA | 121 | 38 | 33 | 50 | 0.84 | (0.52-1.35) | 0.473 | | 0.81 | (0.48-1.37) | | | 0.433 | | 0.88 | (0.55-1.39) | 0.573 | |
| *TNF* | GG | 373 | 110 | 99 | 164 |  |  |  | |  |  | | |  | |  |  |  | |
| rs361525 | GA | 18 | 4 | 6 | 8 | 1.56 | (0.5-4.91) | 0.447 | | 1.41 | (0.4-4.94) | | | 0.595 | |  |  |  | |
|  | AA | 1 | 0 | 0 | 1 | 0.00 | (0-0) | 0.000 | | 0.00 | (0-0) | | | 0.000 | |  |  |  | |
|  | GA/AA | 19 | 4 | 6 | 9 | 1.68 | (0.54-5.22) | 0.373 | | 1.63 | (0.48-5.56) | | | 0.437 | | 1.60 | (0.52-4.93) | 0.411 | |
| *TNFAIP3* | CC | 223 | 64 | 60 | 99 |  |  |  | |  |  | | |  | |  |  |  | |
| rs6927172 | CG | 141 | 45 | 34 | 62 | 0.85 | (0.54-1.35) | 0.496 | | 0.89 | (0.54-1.48) | | | 0.656 | |  |  |  | |
|  | GG | 26 | 5 | 11 | 10 | 1.75 | (0.63-4.88) | 0.284 | | 1.39 | (0.44-4.37) | | | 0.572 | |  |  |  | |
|  | CG/GG | 167 | 50 | 45 | 72 | 0.94 | (0.6-1.47) | 0.786 | | 0.94 | (0.57-1.53) | | | 0.801 | | 0.91 | (0.59-1.4) | 0.662 | |
| *TNFRSF1A* | TT | 136 | 45 | 34 | 57 |  |  |  | |  |  | | |  | |  |  |  | |
| rs1800693 | TC | 191 | 54 | 53 | 84 | 1.23 | (0.76-1.99) | 0.398 | | 1.16 | (0.68-1.97) | | | 0.582 | |  |  |  | |
|  | CC | 65 | 16 | 19 | 30 | 1.48 | (0.75-2.91) | 0.254 | | 1.36 | (0.65-2.84) | | | 0.419 | |  |  |  | |
|  | TC/CC | 256 | 70 | 72 | 114 | 1.29 | (0.82-2.03) | 0.276 | | 1.21 | (0.73-1.99) | | | 0.467 | | 1.39 | (0.89-2.16) | 0.144 | |
| *TNFRSF1A* | GG | 137 | 33 | 40 | 64 |  |  |  | |  |  | | |  | |  |  |  | |
| rs4149570 | GT | 196 | 68 | 47 | 81 | 0.59 | (0.36-0.98) | 0.040* | | 0.63 | (0.37-1.09) | | | 0.102 | |  |  |  | |
|  | TT | 56 | 15 | 18 | 23 | 0.89 | (0.43-1.85) | 0.760 | | 0.82 | (0.37-1.82) | | | 0.619 | |  |  |  | |
|  | GT/TT | 252 | 83 | 65 | 104 | 0.65 | (0.4-1.04) | 0.074 | | 0.67 | (0.39-1.13) | | | 0.130 | | 0.64 | (0.4-1.02) | 0.058 | |
| *TNFRSF10A* | GG | 107 | 35 | 26 | 46 |  |  |  | |  |  | | |  | |  |  |  | |
| rs20575 | GC | 184 | 56 | 50 | 78 | 1.14 | (0.68-1.91) | 0.623 | | 1.09 | (0.61-1.93) | | | 0.769 | |  |  |  | |
|  | CC | 99 | 24 | 30 | 45 | 1.54 | (0.83-2.86) | 0.169 | | 1.50 | (0.76-2.95) | | | 0.240 | |  |  |  | |
|  | GC/CC | 283 | 80 | 80 | 123 | 1.26 | (0.78-2.05) | 0.350 | | 1.21 | (0.71-2.07) | | | 0.477 | | 1.23 | (0.76-1.97) | 0.397 | |
| Logistic regression, adjusted for gender, age, HAQ-, DMARD at baseline, CRP, RA diagnosis (seropositive/seronegative). Adj. OR: adjusted odds ratio; Crude OR under assumption of a dominant genetic model. P-value: *<0.05, **<0.01 | | | | | | | | | | | | | | | | | | | |
