# Supplementary material for: Anti-TNF Treatment Response in Rheumatoid Arthritis Patients Is Associated with Genetic Variation in the NLRP3-Inflammasome
Source: PLoS One. 2014 Jun 26;9(6):e100361. doi: 10.1371/journal.pone.0100361 (PMC4072633; doi:10.1371/journal.pone.0100361)
Supplement: Table S3 — Adjusted odds ratio (OR)/coefficient for associations between gene variants and ACR50 and relDAS28 response to anti-TNF treatment. (a. All RA patients, b. Seropositive RA patients). (DOCX) [file pone.0100361.s004.docx]

| **Supplementary Table 3a.**  All RA patients. Adjusted odds ratio (OR)/coefficient for associations between gene variants and ACR50 and relDAS28 response to anti-TNF treatment. | | | | | | | | | | | | |
| --- | --- | --- | --- | --- | --- | --- | --- | --- | --- | --- | --- | --- |
|  |  |  | ACR50 response | |  |  |  |  |  | relDAS28 |  |  |
| GENE  (SNP) | GENOTYPE | Freq. | no | Yes | | Adj. OR | 95% CI | P-VALUE |  | Adj. Coeff. | 95% CI | P-VALUE |
| *CD14* | GG | 153 | 103 | 50 | |  |  |  |  |  |  |  |
| rs2569190 | GA | 255 | 175 | 80 | | 0.94 | (0.61-1.46) | 0.798 |  | 0.01 | (-0.06-0.07) | 0.802 |
|  | AA | 94 | 61 | 33 | | 1.13 | (0.65-1.96) | 0.665 |  | -0.07 | (-0.15-0.02) | 0.119 |
|  | GA/AA | 349 | 236 | 113 | | 0.99 | (0.66-1.50) | 0.968 |  | -0.01 | (-0.07-0.05) | 0.712 |
| *IFNG* | TT | 137 | 93 | 44 | |  |  |  |  |  |  |  |
| rs2430561 | TA | 263 | 173 | 90 | | 1.08 | (0.69-1.68) | 0.749 |  | 0.03 | (-0.03-0.10) | 0.353 |
|  | AA | 114 | 82 | 32 | | 0.81 | (0.47-1.41) | 0.460 |  | -0.02 | (-0.10-0.06) | 0.561 |
|  | TA/AA | 377 | 255 | 122 | | 0.99 | (0.65-1.52) | 0.968 |  | 0.01 | (-0.05-0.08) | 0.645 |
| *IL1B* | GG | 255 | 181 | 74 | |  |  |  |  |  |  |  |
| rs1143623 | GC | 223 | 149 | 74 | | 1.22 | (0.82-1.80) | 0.330 |  | 0.02 | (-0.04-0.08) | 0.489 |
|  | CC | 37 | 20 | 17 | | 2.14 | (1.05-4.35) | 0.037* |  | 0.02 | (-0.09-0.13) | 0.693 |
|  | GC/CC | 260 | 169 | 91 | | 1.32 | (0.91-1.93) | 0.145 |  | 0.02 | (-0.04-0.08) | 0.466 |
| *IL1B* | TT | 209 | 146 | 63 | |  |  |  |  |  |  |  |
| rs1143627 | TC | 239 | 165 | 74 | | 1.05 | (0.70-1.59) | 0.799 |  | 0.00 | (-0.06-0.06) | 0.941 |
|  | CC | 64 | 37 | 27 | | 1.66 | (0.92-3.00) | 0.092 |  | 0.02 | (-0.07-0.12) | 0.590 |
|  | TC/CC | 303 | 202 | 101 | | 1.17 | (0.79-1.71) | 0.434 |  | 0.01 | (-0.05-0.06) | 0.809 |
| *IL1B* | GG | 165 | 108 | 57 | |  |  |  |  |  |  |  |
| rs4848306 | GA | 257 | 175 | 82 | | 0.91 | (0.60-1.39) | 0.662 |  | 0.04 | (-0.02-0.10) | 0.203 |
|  | AA | 91 | 65 | 26 | | 0.77 | (0.44-1.36) | 0.376 |  | 0.00 | (-0.08-0.09) | 0.935 |
|  | GA/AA | 348 | 240 | 108 | | 0.87 | (0.59-1.30) | 0.507 |  | 0.03 | (-0.03-0.09) | 0.307 |
| *IL1RN* | TT | 200 | 141 | 59 | |  |  |  |  |  |  |  |
| rs4251961 | TC | 230 | 146 | 84 | | 1.34 | (0.89-2.02) | 0.165 |  | 0.03 | (-0.03-0.09) | 0.389 |
|  | CC | 80 | 60 | 20 | | 0.75 | (0.41-1.38) | 0.357 |  | -0.03 | (-0.11-0.06) | 0.511 |
|  | TC/CC | 310 | 206 | 104 | | 1.17 | (0.79-1.73) | 0.427 |  | 0.01 | (-0.04-0.07) | 0.657 |
| *IL4R* | AA | 169 | 117 | 52 | |  |  |  |  |  |  |  |
| rs1805010 | AG | 264 | 179 | 85 | | 1.03 | (0.67-1.59) | 0.878 |  | -0.02 | (-0.08-0.04) | 0.497 |
|  | GG | 86 | 57 | 29 | | 1.15 | (0.66-2.01) | 0.625 |  | 0.00 | (-0.08-0.08) | 0.994 |
|  | AG/GG | 350 | 236 | 114 | | 1.06 | (0.71-1.59) | 0.768 |  | -0.02 | (-0.08-0.04) | 0.591 |
| *IL6* | TT | 318 | 220 | 98 | |  |  |  |  |  |  |  |
| rs10499563 | TC | 177 | 114 | 63 | | 1.23 | (0.83-1.83) | 0.301 |  | 0.02 | (-0.04-0.07) | 0.619 |
|  | CC | 22 | 16 | 6 | | 0.92 | (0.35-2.46) | 0.873 |  | 0.07 | (-0.07-0.21) | 0.335 |
|  | TC/CC | 199 | 130 | 69 | | 1.20 | (0.81-1.76) | 0.359 |  | 0.02 | (-0.04-0.08) | 0.475 |
| *IL6R* | CC | 188 | 125 | 63 | |  |  |  |  |  |  |  |
| rs4537545 | CT | 250 | 170 | 80 | | 0.96 | (0.64-1.45) | 0.853 |  | -0.04 | (-0.10-0.02) | 0.198 |
|  | TT | 76 | 55 | 21 | | 0.77 | (0.42-1.39) | 0.386 |  | -0.02 | (-0.10-0.07) | 0.687 |
|  | CT/TT | 326 | 225 | 101 | | 0.91 | (0.62-1.35) | 0.654 |  | -0.03 | (-0.09-0.02) | 0.237 |
| *IL10* | CC | 312 | 215 | 97 | |  |  |  |  |  |  |  |
| rs1800872 | CA | 177 | 115 | 62 | | 1.22 | (0.82-1.81) | 0.329 |  | 0.01 | (-0.05-0.07) | 0.663 |
|  | AA | 26 | 19 | 7 | | 0.77 | (0.31-1.97) | 0.591 |  | 0.03 | (-0.10-0.16) | 0.600 |
|  | CA/AA | 203 | 134 | 69 | | 1.16 | (0.79-1.69) | 0.460 |  | 0.02 | (-0.04-0.07) | 0.583 |
| *IL10* | CC | 350 | 231 | 119 | |  |  |  |  |  |  |  |
| rs3024505 | CT | 156 | 114 | 42 | | 0.69 | (0.45-1.05) | 0.084 |  | -0.01 | (-0.07-0.05) | 0.743 |
|  | TT | 12 | 6 | 6 | | 1.86 | (0.57-6.06) | 0.300 |  | -0.04 | (-0.22-0.15) | 0.694 |
|  | CT/TT | 168 | 120 | 48 | | 0.75 | (0.50-1.12) | 0.159 |  | -0.01 | (-0.07-0.05) | 0.690 |
| *IL17A* | GG | 234 | 163 | 71 | |  |  |  |  |  |  |  |
| rs2275913 | GA | 228 | 149 | 79 | | 1.29 | (0.87-1.92) | 0.211 |  | -0.03 | (-0.08-0.03) | 0.394 |
|  | AA | 55 | 39 | 16 | | 0.98 | (0.51-1.88) | 0.954 |  | 0.02 | (-0.07-0.12) | 0.633 |
|  | GA/AA | 283 | 188 | 95 | | 1.22 | (0.84-1.79) | 0.298 |  | -0.02 | (-0.07-0.04) | 0.573 |
| *IL23R* | GG | 454 | 310 | 144 | |  |  |  |  |  |  |  |
| rs11209026 | GA | 61 | 40 | 21 | | 1.21 | (0.68-2.14) | 0.516 |  | 0.04 | (-0.05-0.12) | 0.395 |
|  | AA | 1 | 0 | 1 | | - | - | - |  | 0.26 | (-0.37-0.89) | 0.415 |
|  | GA/AA | 62 | 40 | 22 | | 1.26 | (0.72-2.22) | 0.417 |  | 0.04 | (-0.04-0.13) | 0.347 |
| *LY96* | CC | 248 | 168 | 80 | |  |  |  |  |  |  |  |
| rs11465996 | CG | 213 | 142 | 71 | | 1.08 | (0.72-1.60) | 0.715 |  | 0.03 | (-0.03-0.09) | 0.328 |
|  | GG | 54 | 39 | 15 | | 0.82 | (0.43-1.59) | 0.562 |  | 0.01 | (-0.08-0.11) | 0.777 |
|  | CG/GG | 267 | 181 | 86 | | 1.02 | (0.70-1.48) | 0.914 |  | 0.03 | (-0.03-0.08) | 0.355 |
| *MAP3K14* | TT | 151 | 104 | 47 | |  |  |  |  |  |  |  |
| rs7222094 | TC | 254 | 171 | 83 | | 1.16 | (0.74-1.80) | 0.519 |  | 0.01 | (-0.06-0.07) | 0.839 |
|  | CC | 106 | 73 | 33 | | 1.08 | (0.63-1.87) | 0.775 |  | -0.04 | (-0.12-0.04) | 0.301 |
|  | TC/CC | 360 | 244 | 116 | | 1.13 | (0.75-1.72) | 0.554 |  | -0.01 | (-0.07-0.05) | 0.802 |
| *NFKB1* | ins/ins | 213 | 142 | 71 | |  |  |  |  |  |  |  |
| rs28362491 | ins/- | 222 | 156 | 66 | | 0.81 | (0.53-1.22) | 0.303 |  | 0.01 | (-0.06-0.07) | 0.853 |
|  | -/- | 78 | 49 | 29 | | 1.16 | (0.67-2.00) | 0.601 |  | 0.02 | (-0.07-0.10) | 0.680 |
|  | ins/- or  -/- | 300 | 205 | 95 | | 0.89 | (0.61-1.30) | 0.548 |  | 0.01 | (-0.05-0.07) | 0.759 |
| *NFKBIA* | TT | 516 | 350 | 166 | |  |  |  |  |  |  |  |
| rs17103265 | T/- | 2 | 1 | 1 | | - | - | - |  | - | - | - |
|  | -/- | 0 | 0 | 0 | | - | - | - |  | - | - | - |
|  | T/- or  -/- | 2 | 1 | 1 | | - | - | - |  | - | - | - |
| *NFKBIA* | GG | 210 | 147 | 63 | |  |  |  |  |  |  |  |
| rs696 | GA | 233 | 155 | 78 | | 1.23 | (0.82-1.85) | 0.324 |  | 0.03 | (-0.03-0.09) | 0.374 |
|  | AA | 67 | 44 | 23 | | 1.36 | (0.75-2.47) | 0.312 |  | 0.02 | (-0.07-0.11) | 0.722 |
|  | GA/AA | 300 | 199 | 101 | | 1.26 | (0.85-1.85) | 0.250 |  | 0.02 | (-0.03-0.08) | 0.393 |
| *NLRP3* | CC | 275 | 183 | 92 | |  |  |  |  |  |  |  |
| rs4612666 | CT | 210 | 147 | 63 | | 0.86 | (0.58-1.27) | 0.434 |  | -0.06 | (-0.12-0.00) | 0.049* |
|  | TT | 31 | 21 | 10 | | 0.97 | (0.44-2.17) | 0.947 |  | -0.04 | (-0.16-0.08) | 0.524 |
|  | CT/TT | 241 | 168 | 73 | | 0.87 | (0.60-1.27) | 0.467 |  | -0.06 | (-0.11-0.00) | 0.050* |
| *PPARG* | CC | 403 | 277 | 126 | |  |  |  |  |  |  |  |
| rs1801282 | CG | 100 | 65 | 35 | | 1.19 | (0.74-1.90) | 0.468 |  | 0.01 | (-0.06-0.08) | 0.849 |
|  | GG | 9 | 7 | 2 | | 0.73 | (0.15-3.58) | 0.695 |  | 0.03 | (-0.18-0.25) | 0.750 |
|  | CG/GG | 109 | 72 | 37 | | 1.15 | (0.73-1.81) | 0.553 |  | 0.01 | (-0.06-0.08) | 0.793 |
| *PTPN22* | GG | 366 | 251 | 115 | |  |  |  |  |  |  |  |
| rs2476601 | GA | 138 | 90 | 48 | | 1.16 | (0.76-1.76) | 0.496 |  | 0.03 | (-0.04-0.09) | 0.435 |
|  | AA | 10 | 5 | 5 | | 1.99 | (0.52-7.60) | 0.312 |  | 0.07 | (-0.14-0.28) | 0.507 |
|  | GA/AA | 148 | 95 | 53 | | 1.20 | (0.79-1.81) | 0.389 |  | 0.03 | (-0.03-0.09) | 0.374 |
| *SUMO4* | TT | 160 | 109 | 51 | |  |  |  |  |  |  |  |
| rs237025 | TC | 240 | 162 | 78 | | 1.05 | (0.68-1.62) | 0.821 |  | -0.03 | (-0.09-0.04) | 0.433 |
|  | CC | 117 | 79 | 38 | | 1.03 | (0.61-1.73) | 0.917 |  | -0.04 | (-0.12-0.04) | 0.303 |
|  | TC/CC | 357 | 241 | 116 | | 1.04 | (0.70-1.56) | 0.836 |  | -0.03 | (-0.09-0.03) | 0.318 |
| *TGFB1* | CC | 246 | 169 | 77 | |  |  |  |  |  |  |  |
| rs1800469 | CT | 231 | 157 | 74 | | 1.04 | (0.70-1.54) | 0.838 |  | 0.03 | (-0.03-0.08) | 0.379 |
|  | TT | 42 | 25 | 17 | | 1.52 | (0.77-3.01) | 0.226 |  | 0.02 | (-0.09-0.12) | 0.728 |
|  | CT/TT | 273 | 182 | 91 | | 1.11 | (0.76-1.61) | 0.593 |  | 0.02 | (-0.03-0.08) | 0.380 |
| *TLR2* | CC | 231 | 157 | 74 | |  |  |  |  |  |  |  |
| rs11938228 | CA | 232 | 160 | 72 | | 0.91 | (0.61-1.36) | 0.659 |  | 0.03 | (-0.03-0.08) | 0.391 |
|  | AA | 56 | 34 | 22 | | 1.51 | (0.82-2.79) | 0.190 |  | 0.01 | (-0.08-0.11) | 0.813 |
|  | CA/AA | 288 | 194 | 94 | | 1.01 | (0.70-1.47) | 0.947 |  | 0.02 | (-0.03-0.08) | 0.421 |
| *TLR2* | CC | 393 | 258 | 135 | |  |  |  |  |  |  |  |
| rs1816702 | CT | 112 | 84 | 28 | | 0.63 | (0.39-1.01) | 0.057 |  | -0.03 | (-0.09-0.04) | 0.410 |
|  | TT | 0 | 0 | 0 | | - | - | - |  | - | - | - |
|  | CT/TT | 112 | 84 | 28 | | 0.63 | (0.39-1.01) | 0.057 |  | -0.03 | (-0.09-0.04) | 0.410 |
| *TLR2* | TT | 167 | 109 | 58 | |  |  |  |  |  |  |  |
| rs3804099 | TC | 250 | 171 | 79 | | 0.84 | (0.55-1.28) | 0.416 |  | 0.00 | (-0.07-0.06) | 0.905 |
|  | CC | 93 | 67 | 26 | | 0.72 | (0.41-1.26) | 0.245 |  | 0.03 | (-0.05-0.11) | 0.491 |
|  | TC/CC | 343 | 238 | 105 | | 0.80 | (0.54-1.20) | 0.287 |  | 0.00 | (-0.06-0.07) | 0.871 |
| *TLR2* | AA | 136 | 88 | 48 | |  |  |  |  |  |  |  |
| rs4696480 | AT | 264 | 183 | 81 | | 0.75 | (0.48-1.17) | 0.202 |  | -0.01 | (-0.07-0.06) | 0.821 |
|  | TT | 116 | 79 | 37 | | 0.86 | (0.50-1.46) | 0.574 |  | -0.01 | (-0.09-0.07) | 0.749 |
|  | AT/TT | 380 | 262 | 118 | | 0.78 | (0.51-1.19) | 0.247 |  | -0.01 | (-0.07-0.05) | 0.772 |
| *TLR4* | TT | 189 | 121 | 68 | |  |  |  |  |  |  |  |
| rs12377632 | TC | 243 | 171 | 72 | | 0.72 | (0.48-1.09) | 0.117 |  | -0.03 | (-0.09-0.03) | 0.331 |
|  | CC | 75 | 54 | 21 | | 0.70 | (0.39-1.27) | 0.238 |  | 0.00 | (-0.08-0.09) | 0.969 |
|  | TC/CC | 318 | 225 | 93 | | 0.71 | (0.48-1.06) | 0.091 |  | -0.02 | (-0.08-0.03) | 0.440 |
| *TLR4* | TT | 286 | 192 | 94 | |  |  |  |  |  |  |  |
| rs1554973 | TC | 201 | 135 | 66 | | 0.98 | (0.66-1.44) | 0.906 |  | -0.02 | (-0.08-0.04) | 0.456 |
|  | CC | 30 | 23 | 7 | | 0.60 | (0.25-1.47) | 0.267 |  | -0.01 | (-0.13-0.11) | 0.898 |
|  | TC/CC | 231 | 158 | 73 | | 0.92 | (0.63-1.34) | 0.675 |  | -0.02 | (-0.08-0.04) | 0.476 |
| *TLR4* | GG | 253 | 183 | 70 | |  |  |  |  |  |  |  |
| rs5030728 | GA | 215 | 134 | 81 | | 1.58 | (1.06-2.35) | 0.023* |  | 0.01 | (-0.05-0.07) | 0.786 |
|  | AA | 46 | 32 | 14 | | 1.21 | (0.61-2.42) | 0.584 |  | 0.01 | (-0.09-0.11) | 0.794 |
|  | GA/AA | 261 | 166 | 95 | | 1.51 | (1.03-2.21) | 0.033* |  | 0.01 | (-0.05-0.06) | 0.748 |
| *TLR5* | CC | 448 | 307 | 141 | |  |  |  |  |  |  |  |
| rs5744168 | CT | 66 | 42 | 24 | | 1.25 | (0.72-2.15) | 0.429 |  | 0.02 | (-0.07-0.10) | 0.715 |
|  | TT | 2 | 1 | 1 | | 2.07 | (0.13-33.81) | 0.608 |  | -0.03 | (-0.48-0.41) | 0.879 |
|  | CT/TT | 68 | 43 | 25 | | 1.27 | (0.74-2.17) | 0.389 |  | 0.01 | (-0.07-0.10) | 0.737 |
| *TLR9* | TT | 188 | 120 | 68 | |  |  |  |  |  |  |  |
| rs187084 | TC | 243 | 166 | 77 | | 0.83 | (0.55-1.25) | 0.371 |  | -0.02 | (-0.08-0.04) | 0.469 |
|  | CC | 87 | 64 | 23 | | 0.66 | (0.37-1.16) | 0.150 |  | -0.03 | (-0.11-0.05) | 0.478 |
|  | TC/CC | 330 | 230 | 100 | | 0.78 | (0.53-1.15) | 0.213 |  | -0.02 | (-0.08-0.03) | 0.405 |
| *TLR9* | GG | 165 | 120 | 45 | |  |  |  |  |  |  |  |
| rs352139 | GA | 233 | 155 | 78 | | 1.32 | (0.84-2.05) | 0.226 |  | -0.01 | (-0.07-0.06) | 0.793 |
|  | AA | 113 | 72 | 41 | | 1.51 | (0.89-2.54) | 0.126 |  | 0.03 | (-0.05-0.10) | 0.507 |
|  | GA/AA | 346 | 227 | 119 | | 1.38 | (0.91-2.08) | 0.132 |  | 0.00 | (-0.06-0.06) | 0.929 |
| *TNF* | GG | 348 | 232 | 116 | |  |  |  |  |  |  |  |
| rs1800629 | GA | 155 | 108 | 47 | | 0.86 | (0.56-1.30) | 0.460 |  | -0.03 | (-0.09-0.03) | 0.283 |
|  | AA | 12 | 8 | 4 | | 1.10 | (0.32-3.84) | 0.881 |  | 0.01 | (-0.18-0.20) | 0.914 |
|  | GA/AA | 167 | 116 | 51 | | 0.87 | (0.58-1.30) | 0.502 |  | -0.03 | (-0.09-0.03) | 0.317 |
| *TNF* | GG | 493 | 334 | 159 | |  |  |  |  |  |  |  |
| rs361525 | GA | 23 | 14 | 9 | | 1.45 | (0.61-3.47) | 0.399 |  | 0.09 | (-0.05-0.22) | 0.194 |
|  | AA | 1 | 1 | 0 | | - | - | - |  | 0.25 | (-0.38-0.88) | 0.443 |
|  | GA/AA | 24 | 15 | 9 | | 1.36 | (0.58-3.22) | 0.480 |  | 0.10 | (-0.04-0.23) | 0.154 |
| *TNFAIP3* | CC | 298 | 204 | 94 | |  |  |  |  |  |  |  |
| rs6927172 | CG | 187 | 125 | 62 | | 1.13 | (0.76-1.68) | 0.551 |  | -0.02 | (-0.08-0.04) | 0.498 |
|  | GG | 31 | 21 | 10 | | 1.05 | (0.47-2.34) | 0.900 |  | 0.00 | (-0.12-0.12) | 0.976 |
|  | CG/GG | 218 | 146 | 72 | | 1.12 | (0.76-1.63) | 0.567 |  | -0.02 | (-0.07-0.04) | 0.549 |
| *TNFRSF1A* | TT | 180 | 120 | 60 | |  |  |  |  |  |  |  |
| rs1800693 | TC | 250 | 172 | 78 | | 0.91 | (0.6-1.38) | 0.666 |  | -0.01 | (-0.07-0.05) | 0.711 |
|  | CC | 88 | 59 | 29 | | 0.97 | (0.56-1.67) | 0.900 |  | 0.01 | (-0.07-0.09) | 0.857 |
|  | TC/CC | 338 | 231 | 107 | | 0.93 | (0.63-1.37) | 0.701 |  | -0.01 | (-0.07-0.05) | 0.822 |
| *TNFRSF1A* | GG | 178 | 118 | 60 | |  |  |  |  |  |  |  |
| rs4149570 | GT | 261 | 180 | 81 | | 0.91 | (0.60-1.38) | 0.659 |  | -0.05 | (-0.11-0.02) | 0.151 |
|  | TT | 76 | 53 | 23 | | 0.87 | (0.48-1.57) | 0.638 |  | -0.03 | (-0.12-0.06) | 0.490 |
|  | GT/TT | 337 | 233 | 104 | | 0.90 | (0.61-1.34) | 0.606 |  | -0.04 | (-0.10-0.02) | 0.162 |
| *TNFRSF10A* | GG | 134 | 90 | 44 | |  |  |  |  |  |  |  |
| rs20575 | GC | 249 | 171 | 78 | | 0.95 | (0.60-1.49) | 0.810 |  | 0.03 | (-0.04-0.10) | 0.396 |
|  | CC | 132 | 90 | 42 | | 0.95 | (0.56-1.60) | 0.850 |  | 0.03 | (-0.05-0.10) | 0.503 |
|  | GC/CC | 381 | 261 | 120 | | 0.95 | (0.62-1.45) | 0.804 |  | 0.03 | (-0.04-0.09) | 0.381 |
| Adj. OR: adjusted odds ratio for ACR50 and coefficient (Coeff.) for relative change in DAS28 (relDAS28). Adjusted for gender, age, HAQ-, DMARD at baseline, CRP, RA diagnosis (seropositive/seronegative). Freq.: frequency | | | | | | | | | | | | |

| **Supplementary Table 3b.** Seropositive RA patients. Adjusted odds ratio (OR) for associations between gene variants and ACR50 and relDAS28 response to anti-TNF treatment. | | | | | | | | | | | | |
| --- | --- | --- | --- | --- | --- | --- | --- | --- | --- | --- | --- | --- |
|  |  |  | ACR50 response | |  |  |  |  |  | relDAS28 |  |  |
| GENE  (SNP) | GENOTYPE | Freq. | no | Yes | | Adj. OR | 95% CI | P-VALUE |  | Adj. Coeff. | 95% CI | P-VALUE |
| *CD14* | GG | 116 | 74 | 42 | |  |  |  |  |  |  |  |
| rs2569190 | GA | 193 | 133 | 60 | | 0.81 | (0.50-1.33) | 0.412 |  | 0.01 | (-0.06-0.09) | 0.708 |
|  | AA | 71 | 47 | 24 | | 0.90 | (0.48-1.71) | 0.748 |  | -0.08 | (-0.18-0.02) | 0.116 |
|  | GA/AA | 264 | 180 | 84 | | 0.84 | (0.52-1.34) | 0.452 |  | -0.01 | (-0.08-0.06) | 0.789 |
| *IFNG* | TT | 106 | 71 | 35 | |  |  |  |  |  |  |  |
| rs2430561 | TA | 205 | 135 | 70 | | 1.04 | (0.63-1.72) | 0.880 |  | 0.06 | (-0.02-0.14) | 0.163 |
|  | AA | 78 | 54 | 24 | | 0.87 | (0.46-1.66) | 0.682 |  | -0.02 | (-0.11-0.08) | 0.764 |
|  | TA/AA | 283 | 189 | 94 | | 0.99 | (0.61-1.61) | 0.976 |  | 0.04 | (-0.04-0.11) | 0.339 |
| *IL1B* | GG | 191 | 136 | 55 | |  |  |  |  |  |  |  |
| rs1143623 | GC | 169 | 109 | 60 | | 1.37 | (0.87-2.15) | 0.170 |  | 0.04 | (-0.03-0.11) | 0.294 |
|  | CC | 30 | 17 | 13 | | 1.90 | (0.85-4.21) | 0.116 |  | 0.01 | (-0.12-0.14) | 0.841 |
|  | GC/CC | 199 | 126 | 73 | | 1.44 | (0.94-2.22) | 0.097 |  | 0.03 | (-0.03-0.10) | 0.323 |
| *IL1B* | TT | 158 | 112 | 46 | |  |  |  |  |  |  |  |
| rs1143627 | TC | 177 | 119 | 58 | | 1.21 | (0.75-1.94) | 0.431 |  | 0.02 | (-0.06-0.09) | 0.661 |
|  | CC | 54 | 30 | 24 | | 1.85 | (0.97-3.53) | 0.064 |  | 0.04 | (-0.07-0.14) | 0.467 |
|  | TC/CC | 231 | 149 | 82 | | 1.34 | (0.86-2.09) | 0.195 |  | 0.02 | (-0.05-0.09) | 0.537 |
| *IL1B* | GG | 130 | 84 | 46 | |  |  |  |  |  |  |  |
| rs4848306 | GA | 198 | 131 | 67 | | 0.99 | (0.62-1.59) | 0.960 |  | 0.07 | (-0.01-0.14) | 0.088 |
|  | AA | 63 | 47 | 16 | | 0.64 | (0.32-1.26) | 0.196 |  | -0.01 | (-0.12-0.09) | 0.792 |
|  | GA/AA | 261 | 178 | 83 | | 0.89 | (0.57-1.40) | 0.622 |  | 0.05 | (-0.03-0.12) | 0.208 |
| *IL1RN* | TT | 149 | 104 | 45 | |  |  |  |  |  |  |  |
| rs4251961 | TC | 178 | 112 | 66 | | 1.30 | (0.82-2.08) | 0.268 |  | 0.03 | (-0.05-0.10) | 0.446 |
|  | CC | 58 | 42 | 16 | | 0.80 | (0.40-1.60) | 0.523 |  | -0.03 | (-0.13-0.07) | 0.568 |
|  | TC/CC | 236 | 154 | 82 | | 1.17 | (0.75-1.83) | 0.493 |  | 0.01 | (-0.06-0.08) | 0.680 |
| *IL4R* | AA | 126 | 86 | 40 | |  |  |  |  |  |  |  |
| rs1805010 | AG | 204 | 136 | 68 | | 1.11 | (0.68-1.81) | 0.670 |  | -0.03 | (-0.11-0.05) | 0.450 |
|  | GG | 64 | 42 | 22 | | 1.18 | (0.62-2.25) | 0.614 |  | 0.00 | (-0.10-0.11) | 0.934 |
|  | AG/GG | 268 | 178 | 90 | | 1.13 | (0.71-1.80) | 0.608 |  | -0.02 | (-0.09-0.05) | 0.570 |
| *IL6* | TT | 243 | 166 | 77 | |  |  |  |  |  |  |  |
| rs10499563 | TC | 135 | 86 | 49 | | 1.24 | (0.78-1.95) | 0.360 |  | 0.00 | (-0.07-0.08) | 0.895 |
|  | CC | 14 | 10 | 4 | | 0.90 | (0.27-2.99) | 0.861 |  | 0.05 | (-0.13-0.24) | 0.563 |
|  | TC/CC | 149 | 96 | 53 | | 1.20 | (0.77-1.87) | 0.416 |  | 0.01 | (-0.06-0.08) | 0.792 |
| *IL6R* | CC | 137 | 93 | 44 | |  |  |  |  |  |  |  |
| rs4537545 | CT | 192 | 126 | 66 | | 1.14 | (0.71-1.83) | 0.582 |  | -0.02 | (-0.10-0.05) | 0.519 |
|  | TT | 59 | 42 | 17 | | 0.85 | (0.43-1.69) | 0.650 |  | -0.01 | (-0.12-0.09) | 0.780 |
|  | CT/TT | 251 | 168 | 83 | | 1.07 | (0.68-1.69) | 0.765 |  | -0.02 | (-0.09-0.05) | 0.536 |
| *IL10* | CC | 236 | 159 | 77 | |  |  |  |  |  |  |  |
| rs1800872 | CA | 132 | 86 | 46 | | 1.10 | (0.70-1.74) | 0.680 |  | -0.02 | (-0.09-0.06) | 0.663 |
|  | AA | 21 | 15 | 6 | | 0.78 | (0.28-2.18) | 0.632 |  | 0.04 | (-0.11-0.20) | 0.583 |
|  | CA/AA | 153 | 101 | 52 | | 1.05 | (0.68-1.64) | 0.815 |  | -0.01 | (-0.08-0.06) | 0.818 |
| *IL10* | CC | 266 | 171 | 95 | |  |  |  |  |  |  |  |
| rs3024505 | CT | 119 | 88 | 31 | | 0.62 | (0.38-1.01) | 0.056 |  | -0.02 | (-0.09-0.06) | 0.654 |
|  | TT | 9 | 5 | 4 | | 1.42 | (0.36-5.54) | 0.613 |  | -0.09 | (-0.31-0.14) | 0.444 |
|  | CT/TT | 128 | 93 | 35 | | 0.66 | (0.41-1.06) | 0.087 |  | -0.02 | (-0.09-0.05) | 0.554 |
| *IL17A* | GG | 176 | 127 | 49 | |  |  |  |  |  |  |  |
| rs2275913 | GA | 172 | 106 | 66 | | 1.73 | (1.09-2.75) | 0.021* |  | 0.01 | (-0.06-0.08) | 0.752 |
|  | AA | 44 | 30 | 14 | | 1.30 | (0.63-2.69) | 0.471 |  | 0.05 | (-0.06-0.16) | 0.363 |
|  | GA/AA | 216 | 136 | 80 | | 1.63 | (1.05-2.54) | 0.030* |  | 0.02 | (-0.05-0.09) | 0.562 |
| *IL23R* | GG | 346 | 232 | 114 | |  |  |  |  |  |  |  |
| rs11209026 | GA | 43 | 29 | 14 | | 1.06 | (0.53-2.10) | 0.872 |  | 0.04 | (-0.07-0.15) | 0.441 |
|  | AA | 1 | 0 | 1 | | - | - | - |  | 0.25 | (-0.42-0.91) | 0.465 |
|  | GA/AA | 44 | 29 | 15 | | 1.13 | (0.57-2.21) | 0.731 |  | 0.05 | (-0.06-0.15) | 0.388 |
| *LY96* | CC | 188 | 122 | 66 | |  |  |  |  |  |  |  |
| rs11465996 | CG | 162 | 111 | 51 | | 0.87 | (0.55-1.37) | 0.553 |  | 0.02 | (-0.06-0.09) | 0.659 |
|  | GG | 41 | 28 | 13 | | 0.88 | (0.42-1.84) | 0.736 |  | 0.02 | (-0.09-0.14) | 0.711 |
|  | CG/GG | 203 | 139 | 64 | | 0.87 | (0.57-1.34) | 0.535 |  | 0.02 | (-0.05-0.08) | 0.615 |
| *MAP3K14* | TT | 117 | 80 | 37 | |  |  |  |  |  |  |  |
| rs7222094 | TC | 192 | 126 | 66 | | 1.16 | (0.71-1.91) | 0.554 |  | 0.01 | (-0.07-0.08) | 0.894 |
|  | CC | 78 | 53 | 25 | | 1.07 | (0.57-2.01) | 0.823 |  | -0.05 | (-0.15-0.04) | 0.273 |
|  | TC/CC | 270 | 179 | 91 | | 1.14 | (0.71-1.82) | 0.595 |  | -0.01 | (-0.09-0.06) | 0.749 |
| *NFKB1* | ATTG/ATTG | 157 | 106 | 51 | |  |  |  |  |  |  |  |
| rs28362491 | ATTG/del | 176 | 119 | 57 | | 0.97 | (0.61-1.54) | 0.891 |  | 0.04 | (-0.04-0.11) | 0.324 |
|  | del/del | 56 | 35 | 21 | | 1.21 | (0.63-2.29) | 0.568 |  | 0.04 | (-0.06-0.15) | 0.423 |
|  | 1xdel | 232 | 154 | 78 | | 1.02 | (0.66-1.58) | 0.920 |  | 0.04 | (-0.03-0.11) | 0.277 |
| *NFKBIA* | T/T | 390 | 261 | 129 | |  |  |  |  |  |  |  |
| rs17103265 | T/del | 2 | 1 | 1 | | - | - | - |  | - | - | - |
|  | del/del |  | 0 | 0 | | - | - | - |  | - | - | - |
|  | 1xdel | 2 | 1 | 1 | | 2.42 | (0.14-40.58) | 0.539 |  | 0.02 | (-0.45-0.48) | 0.948 |
| *NFKBIA* | GG | 159 | 108 | 51 | |  |  |  |  |  |  |  |
| rs696 | GA | 179 | 122 | 57 | | 1.04 | (0.66-1.67) | 0.854 |  | 0.03 | (-0.05-0.10) | 0.485 |
|  | AA | 49 | 29 | 20 | | 1.58 | (0.81-3.08) | 0.182 |  | 0.01 | (-0.09-0.12) | 0.790 |
|  | GA/AA | 228 | 151 | 77 | | 1.15 | (0.74-1.78) | 0.544 |  | 0.02 | (-0.05-0.09) | 0.504 |
| *NLRP3* | CC | 212 | 137 | 75 | |  |  |  |  |  |  |  |
| rs4612666 | CT | 156 | 109 | 47 | | 0.80 | (0.51-1.25) | 0.330 |  | -0.08 | (-0.15--0.01) | 0.024* |
|  | TT | 25 | 17 | 8 | | 0.90 | (0.37-2.19) | 0.813 |  | -0.08 | (-0.22-0.06) | 0.256 |
|  | CT/TT | 181 | 126 | 55 | | 0.81 | (0.53-1.25) | 0.345 |  | -0.08 | (-0.15--0.01) | 0.018* |
| *PPARG* | CC | 307 | 208 | 99 | |  |  |  |  |  |  |  |
| rs1801282 | CG | 74 | 47 | 27 | | 1.19 | (0.69-2.05) | 0.526 |  | 0.00 | (-0.09-0.08) | 0.942 |
|  | GG | 8 | 6 | 2 | | 0.83 | (0.16-4.27) | 0.826 |  | 0.08 | (-0.16-0.32) | 0.498 |
|  | CG/GG | 82 | 53 | 29 | | 1.16 | (0.68-1.95) | 0.589 |  | 0.00 | (-0.08-0.09) | 0.906 |
| *PTPN22* | GG | 269 | 182 | 87 | |  |  |  |  |  |  |  |
| rs2476601 | GA | 113 | 73 | 40 | | 1.16 | (0.72-1.85) | 0.543 |  | 0.02 | (-0.05-0.10) | 0.532 |
|  | AA | 8 | 4 | 4 | | 1.62 | (0.36-7.38) | 0.533 |  | 0.00 | (-0.24-0.25) | 0.974 |
|  | GA/AA | 121 | 77 | 44 | | 1.18 | (0.75-1.86) | 0.476 |  | 0.02 | (-0.05-0.10) | 0.544 |
| *SUMO4* | TT | 124 | 83 | 41 | |  |  |  |  |  |  |  |
| rs237025 | TC | 173 | 112 | 61 | | 1.10 | (0.67-1.80) | 0.698 |  | -0.03 | (-0.11-0.05) | 0.449 |
|  | CC | 94 | 66 | 28 | | 0.84 | (0.46-1.51) | 0.558 |  | -0.07 | (-0.16-0.02) | 0.154 |
|  | TC/CC | 267 | 178 | 89 | | 1.01 | (0.64-1.59) | 0.980 |  | -0.04 | (-0.11-0.03) | 0.247 |
| *TGFB1* | CC | 189 | 126 | 63 | |  |  |  |  |  |  |  |
| rs1800469 | CT | 174 | 121 | 53 | | 0.89 | (0.57-1.39) | 0.600 |  | -0.01 | (-0.08-0.06) | 0.846 |
|  | TT | 31 | 16 | 15 | | 1.93 | (0.88-4.21) | 0.099 |  | 0.04 | (-0.09-0.17) | 0.558 |
|  | CT/TT | 205 | 137 | 68 | | 1.01 | (0.66-1.54) | 0.978 |  | 0.00 | (-0.07-0.07) | 0.997 |
| *TLR2* | CC | 175 | 116 | 59 | |  |  |  |  |  |  |  |
| rs11938228 | CA | 180 | 123 | 57 | | 0.84 | (0.53-1.33) | 0.458 |  | 0.02 | (-0.05-0.09) | 0.570 |
|  | AA | 39 | 24 | 15 | | 1.22 | (0.59-2.52) | 0.591 |  | -0.02 | (-0.13-0.10) | 0.787 |
|  | CA/AA | 219 | 147 | 72 | | 0.90 | (0.59-1.39) | 0.645 |  | 0.01 | (-0.05-0.08) | 0.689 |
| *TLR2* | CC | 293 | 194 | 99 | |  |  |  |  |  |  |  |
| rs1816702 | CT | 87 | 60 | 27 | | 0.85 | (0.51-1.44) | 0.551 |  | 0.02 | (-0.05-0.09) | 0.565 |
|  | TT |  | 0 | 0 | | - | - | - |  | - | - | - |
|  | CT/TT | 87 | 60 | 27 | | 0.85 | (0.51-1.44) | 0.551 |  | 0.02 | (-0.05-0.09) | 0.565 |
| *TLR2* | TT | 121 | 77 | 44 | |  |  |  |  |  |  |  |
| rs3804099 | TC | 191 | 129 | 62 | | 0.84 | (0.52-1.37) | 0.491 |  | -0.01 | (-0.09-0.07) | 0.833 |
|  | CC | 75 | 54 | 21 | | 0.73 | (0.38-1.37) | 0.325 |  | 0.03 | (-0.06-0.13) | 0.500 |
|  | TC/CC | 266 | 183 | 83 | | 0.81 | (0.51-1.29) | 0.370 |  | 0.00 | (-0.07-0.08) | 0.929 |
| *TLR2* | AA | 102 | 67 | 35 | |  |  |  |  |  |  |  |
| rs4696480 | AT | 202 | 136 | 66 | | 0.82 | (0.49-1.37) | 0.440 |  | 0.00 | (-0.08-0.08) | 0.990 |
|  | TT | 89 | 59 | 30 | | 0.90 | (0.49-1.65) | 0.729 |  | -0.01 | (-0.10-0.09) | 0.905 |
|  | AT/TT | 291 | 195 | 96 | | 0.84 | (0.52-1.37) | 0.486 |  | 0.00 | (-0.08-0.08) | 0.970 |
| *TLR4* | TT | 147 | 90 | 57 | |  |  |  |  |  |  |  |
| rs12377632 | TC | 189 | 133 | 56 | | 0.64 | (0.40-1.02) | 0.061 |  | -0.03 | (-0.10-0.04) | 0.400 |
|  | CC | 50 | 36 | 14 | | 0.59 | (0.29-1.21) | 0.152 |  | -0.02 | (-0.13-0.09) | 0.708 |
|  | TC/CC | 239 | 169 | 70 | | 0.63 | (0.41-0.98) | 0.042* |  | -0.03 | (-0.10-0.04) | 0.412 |
| *TLR4* | TT | 211 | 140 | 71 | |  |  |  |  |  |  |  |
| rs1554973 | TC | 156 | 104 | 52 | | 0.98 | (0.63-1.52) | 0.917 |  | -0.02 | (-0.09-0.05) | 0.546 |
|  | CC | 25 | 18 | 7 | | 0.76 | (0.30-1.91) | 0.554 |  | -0.03 | (-0.17-0.11) | 0.710 |
|  | TC/CC | 181 | 122 | 59 | | 0.94 | (0.62-1.45) | 0.792 |  | -0.02 | (-0.09-0.05) | 0.516 |
| *TLR4* | GG | 186 | 133 | 53 | |  |  |  |  |  |  |  |
| rs5030728 | GA | 174 | 108 | 66 | | 1.55 | (0.99-2.43) | 0.056 |  | -0.01 | (-0.08-0.06) | 0.755 |
|  | AA | 32 | 22 | 10 | | 1.21 | (0.53-2.74) | 0.651 |  | 0.03 | (-0.10-0.15) | 0.679 |
|  | GA/AA | 206 | 130 | 76 | | 1.49 | (0.97-2.30) | 0.070 |  | -0.01 | (-0.07-0.06) | 0.880 |
| *TLR5* | CC | 340 | 229 | 111 | |  |  |  |  |  |  |  |
| rs5744168 | CT | 50 | 32 | 18 | | 1.17 | (0.63-2.20) | 0.616 |  | 0.00 | (-0.10-0.10) | 0.985 |
|  | TT | 2 | 1 | 1 | | 2.18 | (0.13-35.46) | 0.583 |  | -0.03 | (-0.51-0.44) | 0.886 |
|  | CT/TT | 52 | 33 | 19 | | 1.20 | (0.65-2.23) | 0.555 |  | 0.00 | (-0.10-0.10) | 0.964 |
| *TLR9* | TT | 137 | 83 | 54 | |  |  |  |  |  |  |  |
| rs187084 | TC | 189 | 129 | 60 | | 0.73 | (0.46-1.16) | 0.186 |  | -0.05 | (-0.13-0.02) | 0.151 |
|  | CC | 67 | 50 | 17 | | 0.56 | (0.29-1.08) | 0.082 |  | -0.04 | (-0.14-0.06) | 0.394 |
|  | TC/CC | 256 | 179 | 77 | | 0.68 | (0.44-1.06) | 0.090 |  | -0.05 | (-0.12-0.02) | 0.150 |
| *TLR9* | GG | 126 | 90 | 36 | |  |  |  |  |  |  |  |
| rs352139 | GA | 182 | 122 | 60 | | 1.22 | (0.74-2.02) | 0.439 |  | 0.01 | (-0.07-0.08) | 0.896 |
|  | AA | 81 | 49 | 32 | | 1.59 | (0.87-2.89) | 0.130 |  | 0.03 | (-0.06-0.12) | 0.534 |
|  | GA/AA | 263 | 171 | 92 | | 1.33 | (0.83-2.12) | 0.237 |  | 0.01 | (-0.06-0.08) | 0.726 |
| *TNF* | GG | 270 | 175 | 95 | |  |  |  |  |  |  |  |
| rs1800629 | GA | 116 | 82 | 34 | | 0.75 | (0.46-1.21) | 0.238 |  | -0.03 | (-0.11-0.04) | 0.354 |
|  | AA | 5 | 4 | 1 | | 0.42 | (0.05-3.93) | 0.450 |  | -0.06 | (-0.36-0.24) | 0.709 |
|  | GA/AA | 121 | 86 | 35 | | 0.73 | (0.46-1.18) | 0.200 |  | -0.04 | (-0.11-0.04) | 0.334 |
| *TNF* | GG | 373 | 248 | 125 | |  |  |  |  |  |  |  |
| rs361525 | GA | 18 | 12 | 6 | | 1.03 | (0.37-2.83) | 0.961 |  | 0.07 | (-0.09-0.23) | 0.408 |
|  | AA | 1 | 1 | 0 | | - | - | - |  | 0.23 | (-0.43-0.90) | 0.486 |
|  | GA/AA | 19 | 13 | 6 | | 0.95 | (0.35-2.60) | 0.927 |  | 0.08 | (-0.08-0.23) | 0.335 |
| *TNFAIP3* | CC | 223 | 150 | 73 | |  |  |  |  |  |  |  |
| rs6927172 | CG | 141 | 93 | 48 | | 1.08 | (0.69-1.70) | 0.728 |  | -0.02 | (-0.09-0.05) | 0.553 |
|  | GG | 26 | 18 | 8 | | 0.96 | (0.40-2.33) | 0.930 |  | 0.04 | (-0.10-0.17) | 0.608 |
|  | CG/GG | 167 | 111 | 56 | | 1.06 | (0.69-1.64) | 0.778 |  | -0.01 | (-0.08-0.06) | 0.714 |
| *TNFRSF1A* | TT | 136 | 90 | 46 | |  |  |  |  |  |  |  |
| rs1800693 | TC | 191 | 130 | 61 | | 0.89 | (0.56-1.44) | 0.644 |  | -0.02 | (-0.09-0.05) | 0.589 |
|  | CC | 65 | 42 | 23 | | 1.06 | (0.57-2.00) | 0.849 |  | 0.03 | (-0.07-0.13) | 0.540 |
|  | TC/CC | 256 | 172 | 84 | | 0.94 | (0.60-1.46) | 0.769 |  | -0.01 | (-0.08-0.06) | 0.837 |
| *TNFRSF1A* | GG | 137 | 87 | 50 | |  |  |  |  |  |  |  |
| rs4149570 | GT | 196 | 137 | 59 | | 0.77 | (0.48-1.23) | 0.268 |  | -0.08 | (-0.15--0.01) | 0.034* |
|  | TT | 56 | 38 | 18 | | 0.81 | (0.41-1.60) | 0.544 |  | -0.04 | (-0.14-0.07) | 0.506 |
|  | GT/TT | 252 | 175 | 77 | | 0.78 | (0.50-1.21) | 0.267 |  | -0.07 | (-0.14-0.00) | 0.051 |
| *TNFRSF10A* | GG | 107 | 70 | 37 | |  |  |  |  |  |  |  |
| rs20575 | GC | 184 | 126 | 58 | | 0.85 | (0.51-1.42) | 0.540 |  | 0.04 | (-0.04-0.12) | 0.343 |
|  | CC | 99 | 66 | 33 | | 0.93 | (0.52-1.66) | 0.801 |  | 0.04 | (-0.06-0.13) | 0.441 |
|  | GC/CC | 283 | 192 | 91 | | 0.88 | (0.55-1.41) | 0.592 |  | 0.04 | (-0.04-0.11) | 0.321 |
| Adj. OR: adjusted odds ratio for ACR50 and coefficient (Coeff.) for relative change in DAS28 (relDAS28). Adjusted for gender, age, HAQ-, DMARD at baseline, CRP, RA diagnosis (seropositive/seronegative). Freq.: frequency | | | | | | | | | | | | |
