# Supplementary material for: Anti-TNF Treatment Response in Rheumatoid Arthritis Patients Is Associated with Genetic Variation in the NLRP3-Inflammasome
Source: PLoS One. 2014 Jun 26;9(6):e100361. doi: 10.1371/journal.pone.0100361 (PMC4072633; doi:10.1371/journal.pone.0100361)
Supplement: Table S4 — Smoking strata: Odds ratio for minor allele carriers achieving EULAR good/moderate response. (DOCX) [file pone.0100361.s005.docx]

| **Supplementary Table 4.** Smoking strata: Odds ratio for minor allele carriers achieving EULAR good/moderate response | | | | | | | | | | | |
| --- | --- | --- | --- | --- | --- | --- | --- | --- | --- | --- | --- |
|  |  |  | All |  |  | Seropositive | |  | |  |  |
|  | Group | No. | OR | 95% CI | P-value | OR | 95% CI | | p-value | |  |
| *NLRP3* | All | 428 | 0.63 | (0.41-0.96) | 0.032* | 0.56 | (0.35-0.92) | | 0.021* | |  |
| (rs4612666) C>T | Ever smoker | 279 | 0.39 | (0.22-0.67) | 0.001** | 0.39 | (0.21-0.71) | | 0.002** | |  |
|  | Current smoker | 135 | 0.24 | (0.10-0.56) | 0.001** | 0.27 | (0.11-0.67) | | 0.005** | |  |
|  | Previous/never | 293 | 0.94 | (0.56-1.59) | 0.825 | 0.79 | (0.43-1.45) | | 0.448 | |  |
|  | never smoker | 149 | 1.50 | (0.72-3.10) | 0.278 | 1.18 | (0.50-2.82) | | 0.703 | |  |
| *IL4R* | All | 431 | 1.18 | (0.75-1.86) | 0.464 | 1.06 | (0.63-1.79) | | 0.828 | |  |
| (rs1805010) | Ever smoker | 281 | 1.40 | (0.79-2.49) | 0.246 | 1.21 | (0.63-2.31) | | 0.564 | |  |
| A>G | Current smoker | 135 | 2.69 | (1.11-6.50) | 0.028* | 2.19 | (0.82-5.86) | | 0.118 | |  |
|  | Previous/never | 296 | 0.85 | (0.49-1.49) | 0.576 | 0.76 | (0.39-1.47) | | 0.419 | |  |
|  | never smoker | 150 | 0.89 | (0.41-1.93) | 0.777 | 0.84 | (0.33-2.12) | | 0.714 | |  |
| *IL17A* | All | 429 | 0.76 | (0.50-1.17) | 0.215 | 1.01 | (0.62-1.64) | | 0.975 | |  |
| (rs2275913) G>A | Ever smoker | 281 | 0.75 | (0.44-1.28) | 0.291 | 0.95 | (0.53-1.73) | | 0.880 | |  |
|  | Current smoker | 134 | 0.78 | (0.36-1.70) | 0.532 | 0.67 | (0.27-1.62) | | 0.370 | |  |
|  | Previous/never | 295 | 0.79 | (0.47-1.33) | 0.370 | 1.23 | (0.67-2.26) | | 0.495 | |  |
|  | never smoker | 148 | 0.80 | (0.38-1.68) | 0.551 | 1.15 | (0.48-2.76) | | 0.753 | |  |
| *TLR2* | All | 418 | 1.08 | (0.64-1.80) | 0.780 | 1.54 | (0.84-2.81) | | 0.161 | |  |
| (rs1816702) C>A | Ever smoker | 270 | 1.04 | (0.56-1.95) | 0.897 | 1.12 | (0.56-2.22) | | 0.747 | |  |
|  | Current smoker | 130 | 0.73 | (0.31-1.71) | 0.465 | 0.71 | (0.29-1.76) | | 0.460 | |  |
|  | Previous/never | 288 | 1.54 | (0.77-3.06) | 0.219 | 3.75 | (1.39-10.12) | | 0.009** | |  |
|  | never smoker | 148 | 1.10 | (0.44-2.76) | 0.846 | 4.45 | (0.95-20.89) | | 0.058 | |  |
| *TGFB1* | All | 431 | 1.04 | (0.68-1.59) | 0.848 | 0.93 | (0.57-1.51) | | 0.764 | |  |
| (rs1800469) C>T | Ever smoker | 281 | 1.37 | (0.81-2.34) | 0.242 | 1.14 | (0.63-2.07) | | 0.657 | |  |
|  | Current smoker | 134 | 1.33 | (0.61-2.89) | 0.468 | 0.84 | (0.35-2.00) | | 0.692 | |  |
|  | Previous/never | 297 | 0.91 | (0.54-1.53) | 0.714 | 0.97 | (0.53-1.77) | | 0.909 | |  |
|  | never smoker | 150 | 0.63 | (0.30-1.36) | 0.240 | 0.62 | (0.25-1.56) | | 0.311 | |  |
| *IL10* | All | 427 | 1.28 | (0.82-1.98) | 0.279 | 1.11 | (0.67-1.83) | | 0.694 | |  |
| (rs1800872) C>A | Ever smoker | 279 | 1.17 | (0.68-2.03) | 0.571 | 1.09 | (0.59-1.99) | | 0.793 | |  |
|  | Current smoker | 133 | 1.93 | (0.85-4.37) | 0.117 | 1.72 | (0.71-4.19) | | 0.230 | |  |
|  | Previous/never | 294 | 1.07 | (0.63-1.82) | 0.808 | 0.88 | (0.47-1.62) | | 0.673 | |  |
|  | never smoker | 148 | 1.39 | (0.64-2.98) | 0.405 | 1.01 | (0.40-2.55) | | 0.988 | |  |
| Logistic regression on RA patient with smoking data adjusted for gender, age, HAQ-score, DMARD at baseline, CRP and RA diagnosis. P-value: *<0.05, **<0.01 | | | | | | | | | | | |
