# Supplementary material for: Anti-TNF Treatment Response in Rheumatoid Arthritis Patients Is Associated with Genetic Variation in the NLRP3-Inflammasome
Source: PLoS One. 2014 Jun 26;9(6):e100361. doi: 10.1371/journal.pone.0100361 (PMC4072633; doi:10.1371/journal.pone.0100361)
Supplement: Table S5 — Anti-tumour necrosis factor (TNF) subgroup odds ratio for EULAR good/moderate response. (a. All RA patients, b. Seropositive RA patients). (DOCX) [file pone.0100361.s006.docx]

**Supplementary Table 5a.**

Anti-tumour necrosis factor (TNF) subgroup odds ratio for EULAR good/moderate response, all RA patients

| GENE | | GENETIC VARIANT | WT/VAR | INFLIX  OR(95 % CI), P-VALUE | ETAN  OR(95 % CI), P-VALUE | ADALIM  OR(95 % CI), P-VALUE | MONOCLONAL AB  OR(95 % CI), P-VALUE | |
| --- | --- | --- | --- | --- | --- | --- | --- | --- |
| CD14 | | rs2569190 | G/A | 0.71(0.34-1.48), 0.365 | 1.56(0.73-3.33), 0.247 | 2.67(0.97-7.31), 0.056 | 1.06(0.64-1.78), 0.812 | |
| IFNG | | rs2430561 | T/A | 0.85(0.41-1.76), 0.661 | 0.61(0.25-1.49), 0.276 | 0.40(0.12-1.39), 0.151 | 0.78(0.46-1.34), 0.372 | |
| IL1B | | rs1143623 | G/C | 0.74(0.38-1.44), 0.374 | 1.98(0.96-4.05), 0.063 | 0.90(0.36-2.25), 0.824 | 0.72(0.44-1.16), 0.178 | |
| IL1B | | rs1143627 | T/C | 0.55(0.28-1.08), 0.084 | 1.51(0.75-3.05), 0.246 | 0.74(0.29-1.88), 0.523 | 0.88(0.55-1.40), 0.576 | |
| IL1B | | rs4848306 | G/A | 2.84(1.39-5.79), 0.004 | 0.83(0.39-1.77), 0.626 | 0.55(0.18-1.64), 0.282 | 1.34(0.82-2.20), 0.246 | |
| IL1RN | | rs4251961 | T/C | 1.46(0.74-2.91), 0.277 | 1.13(0.55-2.31), 0.735 | 0.47(0.17-1.28), 0.139 | 0.95(0.59-1.54), 0.842 | |
| IL4R | | rs1805010 | A/G | 1.48(0.75-2.94), 0.262 | 0.77(0.36-1.67), 0.507 | 1.10(0.42-2.88), 0.842 | 1.22(0.75-2.00), 0.420 | |
| IL6 | | rs10499563 | T/C | 1.51(0.73-3.11), 0.264 | 0.90(0.43-1.85), 0.765 | 1.12(0.43-2.93), 0.820 | 1.26(0.77-2.06), 0.355 | |
| IL6R | | rs4537545 | C/T | 0.90(0.47-1.75), 0.759 | 0.76(0.36-1.62), 0.480 | 1.89(0.73-4.91), 0.190 | 1.05(0.64-1.70), 0.857 | |
| IL10 | | rs1800872 | C/A | 1.56(0.76-3.18), 0.225 | 1.23(0.60-2.52), 0.569 | 0.74(0.29-1.85), 0.518 | 1.33(0.82-2.18), 0.250 | |
| IL10 | | rs3024505 | C/T | 1.22(0.57-2.61), 0.603 | 0.79(0.39-1.60), 0.505 | 1.01(0.38-2.64), 0.990 | 1.09(0.65-1.83), 0.740 | |
| IL17A | | rs2275913 | G/A | 1.02(0.52-1.98), 0.962 | 0.52(0.25-1.07), 0.076 | 0.83(0.33-2.10), 0.699 | 0.93(0.58-1.49), 0.763 | |
| IL23R | | rs11209026 | G/A | 4.54(0.97-21.17), 0.054 | 0.82(0.32-2.13), 0.685 | 0.35(0.11-1.12), 0.077 | 1.40(0.63-3.11), 0.405 | |
| LY96 | | rs11465996 | C/G | 2.20(1.11-4.36), 0.023 | 0.62(0.30-1.30), 0.206 | 0.68(0.28-1.69), 0.409 | 1.28(0.80-2.04), 0.306 | |
| MAP3K14 | | rs7222094 | T/C | 0.89(0.43-1.85), 0.751 | 0.82(0.37-1.79), 0.615 | 1.38(0.54-3.56), 0.504 | 1.01(0.60-1.69), 0.985 | |
| NFKB1 | | rs28362491 | ATTG/del | 0.89(0.45-1.76), 0.747 | 1.65(0.80-3.40), 0.172 | 0.63(0.24-1.61), 0.330 | 0.66(0.40-1.07), 0.092 | |
| NFKBIA | | rs17103265 | T/del | - | - | - | - | |
| NFKBIA | | rs696 | G/A | 1.11(0.57-2.19), 0.756 | 0.88(0.42-1.83), 0.738 | 1.23(0.49-3.08), 0.663 | 1.30(0.81-2.09), 0.278 | |
| NLRP3 | | rs4612666 | C/T | 0.50(0.25-0.97), 0.041 | 0.56(0.27-1.15), 0.112 | 0.99(0.38-2.55), 0.982 | 0.67(0.42-1.08), 0.100 | |
| PPARG | | rs1801282 | C/G | 1.35(0.61-3.00), 0.458 | 1.19(0.49-2.91), 0.705 | 0.74(0.26-2.12), 0.572 | 0.92(0.52-1.61), 0.759 | |
| PTPN22 | | rs2476601 | G/A | 1.35(0.63-2.88), 0.436 | 0.83(0.40-1.76), 0.633 | 1.32(0.42-4.15), 0.630 | 1.12(0.65-1.92), 0.683 | |
| SUMO4 | | rs237025 | T/C | 0.68(0.33-1.40), 0.292 | 0.92(0.42-1.97), 0.821 | 1.19(0.46-3.05), 0.721 | 0.81(0.49-1.35), 0.424 | |
| TGFB1 | | rs1800469 | C/T | 1.56(0.79-3.08), 0.198 | 0.81(0.41-1.64), 0.564 | 0.97(0.39-2.38), 0.941 | 1.14(0.72-1.82), 0.580 | |
| TLR2 | | rs11938228 | C/A | 1.15(0.59-2.24), 0.672 | 0.72(0.35-1.50), 0.386 | 1.85(0.75-4.55), 0.182 | 1.27(0.79-2.02), 0.321 | |
| TLR2 | | rs1816702 | C/T | 0.84(0.38-1.84), 0.660 | 1.29(0.49-3.39), 0.599 | 1.29(0.41-4.09), 0.666 | 0.76(0.44-1.31), 0.329 | |
| TLR2 | | rs3804099 | T/C | 1.07(0.51-2.26), 0.852 | 0.93(0.45-1.92), 0.848 | 2.04(0.78-5.35), 0.146 | 1.34(0.81-2.24), 0.259 | |
| TLR2 | | rs4696480 | T/A | 1.54(0.73-3.26), 0.261 | 0.63(0.26-1.49), 0.291 | 1.89(0.75-4.79), 0.179 | 1.23(0.74-2.06), 0.420 | |
| TLR4 | | rs12377632 | T/C | 1.09(0.55-2.16), 0.805 | 0.81(0.38-1.70), 0.575 | 0.80(0.29-2.20), 0.672 | 0.90(0.55-1.48), 0.687 | |
| TLR4 | | rs1554973 | T/C | 0.77(0.40-1.50), 0.445 | 1.26(0.60-2.63), 0.536 | 1.64(0.64-4.19), 0.304 | 1.04(0.65-1.67), 0.860 | |
| TLR4 | | rs5030728 | G/A | 1.44(0.74-2.79), 0.279 | 0.82(0.40-1.68), 0.582 | 0.94(0.38-2.33), 0.899 | 1.18(0.73-1.89), 0.499 | |
| TLR5 | | rs5744168 | C/T | 0.88(0.36-2.12), 0.768 | 0.89(0.31-2.57), 0.832 | 0.41(0.10-1.61), 0.200 | 0.84(0.43-1.64), 0.615 | |
| TLR9 | | rs187084 | T/C | 1.14(0.58-2.25), 0.706 | 0.80(0.39-1.68), 0.562 | 0.56(0.20-1.59), 0.275 | 0.92(0.56-1.50), 0.737 | |
| TLR9 | | rs352139 | G/A | 0.94(0.46-1.91), 0.857 | 1.08(0.52-2.24), 0.845 | 2.39(0.92-6.17), 0.073 | 1.24(0.75-2.04), 0.398 | |
| TNF | | rs1800629 | G/A | 0.55(0.25-1.20), 0.135 | 0.58(0.28-1.20), 0.143 | 2.16(0.75-6.21), 0.152 | 0.94(0.57-1.53), 0.793 | |
| TNF | | rs361525 | G/A | 2.45(0.26-22.74), 0.432 | 2.21(0.42-11.57), 0.347 | 1.14(0.12-10.59), 0.911 | 1.52(0.41-5.66), 0.532 | |
| TNFAIP3 | | rs6927172 | C/G | 1.30(0.66-2.56), 0.441 | 1.19(0.58-2.43), 0.634 | 0.60(0.24-1.49), 0.269 | 0.95(0.59-1.53), 0.838 | |
| TNFRSF1A | | rs1800693 | A/G | 1.22(0.61-2.44), 0.570 | 1.09(0.51-2.32), 0.823 | 1.26(0.51-3.15), 0.619 | 1.16(0.72-1.88), 0.540 | |
| TNFRSF1A | | rs4149570 | G/T | 1.14(0.56-2.32), 0.718 | 0.40(0.18-0.89), 0.025 | 0.93(0.36-2.39), 0.874 | 0.99(0.60-1.64), 0.974 | |
| TNFRSF10A | | rs20575 | G/C | 1.64(0.79-3.39), 0.185 | 1.60(0.73-3.47), 0.238 | 0.80(0.26-2.44), 0.696 | 1.01(0.59-1.72), 0.972 | |
| OR: adjusted odds ratio for EULAR good and moderate response vs no response. Adjusted for gender, age, HAQ-, DMARD at baseline, CRP, RA diagnosis (seropositive/seronegative). | | | | | | |  |  |

**Supplementary Table 5b.**

Anti-tumour necrosis factor (TNF) subgroup odds ratio for EULAR good/moderate response, seropositive RA patients

| GENE | GENETIC VARIANT | WT/VAR | INFLIX  OR(95 % CI), P-VALUE | ETAN  OR(95 % CI), P-VALUE | ADALIM  OR(95 % CI), P-VALUE | MONOCLONAL AB  OR(95 % CI), P-VALUE |
| --- | --- | --- | --- | --- | --- | --- |
| CD14 | rs2569190 | G/A | 0.63(0.26-1.55), 0.315 | 1.47(0.62-3.49), 0.380 | 3.23(1.00-10.46), 0.050 | 1.06(0.59-1.91), 0.852 |
| IFNG | rs2430561 | T/A | 0.83(0.36-1.93), 0.671 | 0.59(0.21-1.69), 0.325 | 0.53(0.14-1.98), 0.343 | 0.78(0.42-1.44), 0.433 |
| IL1B | rs1143623 | G/C | 0.97(0.45-2.10), 0.946 | 1.80(0.80-4.08), 0.158 | 1.36(0.46-4.03), 0.582 | 1.03(0.60-1.76), 0.918 |
| IL1B | rs1143627 | T/C | 0.76(0.35-1.64), 0.482 | 1.49(0.66-3.35), 0.334 | 0.86(0.28-2.63), 0.785 | 0.84(0.48-1.46), 0.535 |
| IL1B | rs4848306 | G/A | 2.08(0.91-4.73), 0.081 | 1.32(0.56-3.10), 0.521 | 0.61(0.17-2.19), 0.452 | 1.06(0.60-1.88), 0.840 |
| IL1RN | rs4251961 | T/C | 1.95(0.87-4.39), 0.105 | 1.16(0.50-2.69), 0.733 | 0.39(0.11-1.39), 0.147 | 1.04(0.60-1.82), 0.885 |
| IL4R | rs1805010 | A/G | 1.74(0.77-3.91), 0.184 | 0.67(0.27-1.66), 0.384 | 0.67(0.19-2.31), 0.521 | 1.14(0.65-2.01), 0.653 |
| IL6 | rs10499563 | T/C | 0.99(0.43-2.29), 0.982 | 1.06(0.45-2.53), 0.889 | 1.62(0.50-5.28), 0.420 | 1.14(0.65-2.01), 0.643 |
| IL6R | rs4537545 | C/T | 1.07(0.50-2.29), 0.869 | 0.72(0.29-1.75), 0.467 | 2.32(0.73-7.37), 0.152 | 1.21(0.69-2.11), 0.510 |
| IL10 | rs1800872 | C/A | 1.14(0.49-2.65), 0.768 | 1.06(0.46-2.45), 0.883 | 0.51(0.17-1.56), 0.238 | 1.03(0.58-1.80), 0.925 |
| IL10 | rs3024505 | C/T | 1.01(0.42-2.42), 0.977 | 0.98(0.42-2.26), 0.959 | 0.93(0.29-2.99), 0.909 | 0.92(0.51-1.65), 0.779 |
| IL17A | rs2275913 | G/A | 0.93(0.43-2.01), 0.857 | 0.70(0.30-1.61), 0.399 | 1.54(0.52-4.57), 0.434 | 1.14(0.66-1.95), 0.641 |
| IL23R | rs11209026 | G/A | 7.96(0.95-66.52), 0.056 | 1.19(0.37-3.78), 0.773 | 0.19(0.04-0.92), 0.039 | 1.52(0.58-4.02), 0.397 |
| LY96 | rs11465996 | C/G | 1.53(0.70-3.36), 0.283 | 0.38(0.15-0.94), 0.036 | 1.11(0.37-3.37), 0.849 | 1.19(0.70-2.03), 0.521 |
| MAP3K14 | rs7222094 | T/C | 0.60(0.25-1.45), 0.257 | 0.79(0.33-1.91), 0.600 | 2.73(0.90-8.25), 0.076 | 1.06(0.59-1.91), 0.846 |
| NFKB1 | rs28362491 | ATTG/del | 1.07(0.49-2.35), 0.866 | 2.49(1.06-5.81), 0.035 | 0.86(0.29-2.59), 0.795 | 0.73(0.42-1.28), 0.269 |
| NFKBIA | rs17103265 | T/del | - | - | - | - |
| NFKBIA | rs696 | G/A | 1.13(0.51-2.51), 0.759 | 0.99(0.43-2.26), 0.976 | 1.27(0.41-3.89), 0.679 | 1.28(0.74-2.20), 0.380 |
| NLRP3 | rs4612666 | C/T | 0.55(0.26-1.20), 0.132 | 0.42(0.18-0.99), 0.046 | 0.98(0.31-3.11), 0.975 | 0.64(0.37-1.11), 0.113 |
| PPARG | rs1801282 | C/G | 1.98(0.77-5.13), 0.157 | 1.23(0.42-3.63), 0.709 | 0.72(0.22-2.37), 0.585 | 1.06(0.55-2.02), 0.864 |
| PTPN22 | rs2476601 | G/A | 1.55(0.67-3.60), 0.310 | 0.92(0.40-2.11), 0.843 | 1.29(0.33-5.10), 0.718 | 1.12(0.61-2.07), 0.713 |
| SUMO4 | rs237025 | T/C | 0.71(0.30-1.67), 0.433 | 0.93(0.40-2.20), 0.876 | 1.35(0.44-4.07), 0.600 | 0.77(0.43-1.40), 0.399 |
| TGFB1 | rs1800469 | C/T | 1.29(0.59-2.80), 0.524 | 0.62(0.28-1.38), 0.237 | 0.89(0.30-2.60), 0.828 | 0.99(0.58-1.68), 0.959 |
| TLR2 | rs11938228 | C/A | 1.04(0.49-2.21), 0.918 | 0.84(0.36-1.95), 0.685 | 1.30(0.44-3.86), 0.641 | 1.12(0.65-1.91), 0.685 |
| TLR2 | rs1816702 | C/T | 1.17(0.46-2.97), 0.748 | 2.67(0.7-10.17), 0.149 | 2.53(0.45-14.15), 0.29 | 0.95(0.50-1.80), 0.879 |
| TLR2 | rs3804099 | T/C | 0.97(0.41-2.31), 0.945 | 0.67(0.28-1.57), 0.353 | 1.79(0.54-5.90), 0.342 | 1.25(0.69-2.27), 0.470 |
| TLR2 | rs4696480 | T/A | 1.60(0.68-3.79), 0.282 | 0.92(0.34-2.48), 0.870 | 1.98(0.65-6.08), 0.231 | 1.30(0.72-2.33), 0.386 |
| TLR4 | rs12377632 | T/C | 0.96(0.43-2.13), 0.914 | 0.77(0.32-1.85), 0.566 | 0.88(0.28-2.82), 0.836 | 0.80(0.46-1.41), 0.442 |
| TLR4 | rs1554973 | T/C | 0.84(0.39-1.78), 0.641 | 1.49(0.62-3.58), 0.372 | 2.21(0.71-6.87), 0.171 | 1.12(0.65-1.91), 0.680 |
| TLR4 | rs5030728 | G/A | 1.85(0.86-3.98), 0.116 | 0.73(0.31-1.68), 0.458 | 0.72(0.24-2.15), 0.554 | 1.23(0.72-2.11), 0.444 |
| TLR5 | rs5744168 | C/T | 0.97(0.34-2.80), 0.959 | 0.82(0.25-2.70), 0.749 | 0.15(0.03-0.79), 0.025 | 0.80(0.37-1.75), 0.576 |
| TLR9 | rs187084 | T/C | 0.73(0.32-1.64), 0.447 | 0.69(0.29-1.64), 0.403 | 0.63(0.18-2.19), 0.470 | 0.80(0.45-1.42), 0.446 |
| TLR9 | rs352139 | G/A | 1.30(0.58-2.91), 0.531 | 1.15(0.50-2.66), 0.742 | 1.94(0.63-6.02), 0.251 | 1.34(0.76-2.38), 0.307 |
| TNF | rs1800629 | G/A | 0.36(0.14-0.91), 0.030 | 0.59(0.26-1.34), 0.207 | 1.42(0.44-4.65), 0.558 | 1.04(0.57-1.91), 0.887 |
| TNF | rs361525 | G/A | 1.8(0.18-17.99), 0.616 | 1.55(0.26-9.39), 0.632 | - | 1.87(0.39-8.86), 0.432 |
| TNFAIP3 | rs6927172 | C/G | 1.12(0.52-2.42), 0.765 | 1.31(0.58-2.98), 0.519 | 0.82(0.27-2.45), 0.722 | 0.94(0.54-1.64), 0.833 |
| TNFRSF1A | rs1800693 | A/G | 1.77(0.79-3.93), 0.163 | 1.30(0.54-3.14), 0.559 | 1.72(0.58-5.07), 0.324 | 1.42(0.82-2.46), 0.210 |
| TNFRSF1A | rs4149570 | G/T | 0.70(0.30-1.61), 0.398 | 0.32(0.13-0.81), 0.016 | 0.96(0.31-2.95), 0.948 | 0.75(0.42-1.35), 0.336 |
| TNFRSF10A | rs20575 | G/C | 1.70(0.76-3.84), 0.198 | 1.43(0.57-3.59), 0.446 | 1.27(0.38-4.30), 0.699 | 1.17(0.65-2.10), 0.595 |

OR: adjusted odds ratio for EULAR good and moderate response vs no response. Adjusted for gender, age, HAQ-, DMARD at baseline, CRP
